# Cholesterol determines the cytosolic entry and seeded aggregation of tau

**DOI:** 10.1016/j.celrep.2022.110776

**Published:** 2022-05-06

**Authors:** Benjamin J. Tuck, Lauren V.C. Miller, Taxiarchis Katsinelos, Annabel E. Smith, Emma L. Wilson, Sophie Keeling, Shi Cheng, Marina J. Vaysburd, Claire Knox, Lucy Tredgett, Emmanouil Metzakopian, Leo C. James, William A. McEwan

**Affiliations:** 1 UK Dementia Research Institute at the University of Cambridge, Department of Clinical Neurosciences, Hills Road, Cambridge, CB2 0AH, UK; 2 MRC Laboratory of Molecular Biology, Francis Crick Avenue, Cambridge, CB2 0QH, UK

**Keywords:** tau, Alzheimer’s disease, cholesterol, Niemann-Pick disease, seeded aggregation, endocytosis, neurodegeneration

## Abstract

Assemblies of tau can transit between neurons, seeding aggregation in a prion-like manner. To accomplish this, tau must cross cell-limiting membranes, a process that is poorly understood. Here, we establish assays for the study of tau entry into the cytosol as a phenomenon distinct from uptake, in real time, and at physiological concentrations. The entry pathway of tau is cell type specific and, in neurons, highly sensitive to cholesterol. Depletion of the cholesterol transporter Niemann-Pick type C1 or extraction of membrane cholesterol renders neurons highly permissive to tau entry and potentiates seeding even at low levels of exogenous tau assemblies. Conversely, cholesterol supplementation reduces entry and almost completely blocks seeded aggregation. Our findings establish entry as a rate-limiting step to seeded aggregation and demonstrate that dysregulated cholesterol, a feature of several neurodegenerative diseases, potentiates tau aggregation by promoting entry of tau assemblies into the cell interior.

## Highlights

• Cytosolic entry is rate limiting to the seeded aggregation of tau
• Tau assemblies enter the cytosol of neurons via a clathrin-independent pathway
• Low membrane cholesterol renders neurons extremely permissive to entry and seeding
• NPC1 protects against entry and seeded aggregation

## Graphical abstract

**Figure:**
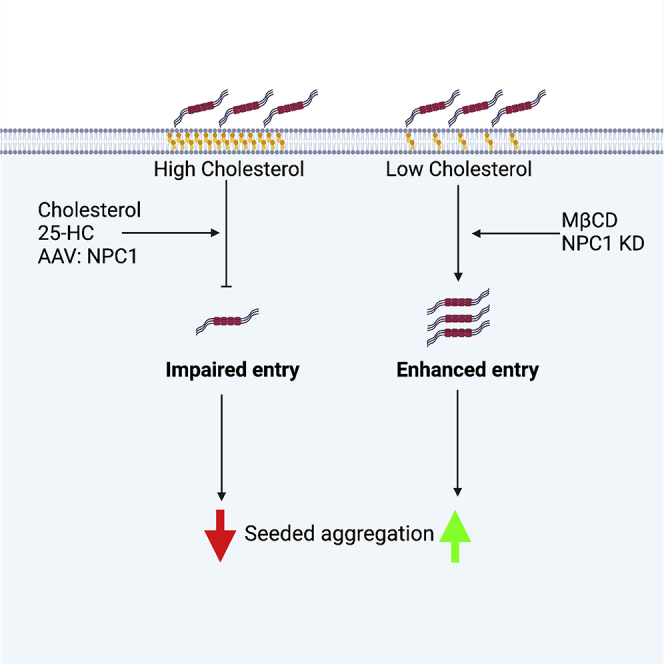

## Teaser

Tuck et al. measure the entry of tau assemblies into the cytosol of neurons with high sensitivity. They identify a critical role for membrane cholesterol in preventing the entry and downstream seeded aggregation of tau. The results provide mechanistic insights into the role of cholesterol in neurodegenerative disease.

## Introduction

Tauopathies are a group of neurodegenerative diseases characterized by conversion of the microtubule-associated protein tau into highly ordered fibrils (Goedert et al., 2017). Most prominent among these diseases is Alzheimer’s disease (AD), which accounts for most dementia cases worldwide. Other tauopathies include frontotemporal dementia, progressive supranuclear palsy, and chronic traumatic encephalopathy. A causal role of tau in neurodegeneration is indicated by more than 50 nonsynonymous and intronic point mutations that lead to dominantly inherited, early-onset forms of neurodegenerative disease characterized by the accumulation of tau assemblies in the brain (Goedert, 2018). Two non-mutually exclusive mechanisms are proposed to explain the occurrence of tau fibrils in the diseased brain. First, nucleation of aggregation may occur in a cell-autonomous manner. Alternatively, tau assemblies may transit between cells, promoting aggregation of tau in recipient cells in a “prion-like” manner. This latter model of propagation could help explain the observed spatiotemporal spread of tau misfolding in the human brain and is consistent with observations of seeded aggregation in cultured cells (Frost et al., 2009; Kfoury et al., 2012; McEwan et al., 2017; Sanders et al., 2014) and in *in vivo* disease models (Clavaguera et al., 2009, 2013; Guo et al., 2016; Iba et al., 2013). The relative contributions of these two mechanisms to human disease progression remain unknown (Mudher et al., 2017). Tau assemblies are taken up into membrane-bound vesicles after interactions between tau and cell-surface heparan sulfate proteoglycans (HSPGs) as well as the recently identified low-density lipoprotein receptor LRP1 (Holmes et al., 2013; Rauch et al., 2020). Tau is then incorporated into membrane-bound compartments via endocytosis and macropinocytosis (Evans et al., 2018; Falcon et al., 2018; Holmes et al., 2013; Wu et al., 2012). For a prion-like mechanism to occur, tau assemblies must gain access to the cytosol, somehow breaching these cell-limiting membranes. Although the process of tau filament uptake has been comparatively well studied, the transfer of tau from intracellular membrane-bound vesicles to the cytosol is largely unexplored and remains a critical missing step for assessing the relevance of seeded aggregation to disease progression (De La-Rocque et al., 2021; Mudher et al., 2017).

Cholesterol is a critical determinant of membrane bilayer structural integrity and a known risk factor in neurodegenerative disease (Arenas et al., 2017; Dai et al., 2021; Notkola et al., 1998; Valenza and Cattaneo, 2006). Cholesterol is depleted from the brain in an age-dependent manner, resulting in impaired intracellular signaling and synaptic plasticity (Egawa et al., 2016; Martín-Segura et al., 2019; Palomer et al., 2016). Variation in *APOE*, which encodes a cholesterol transporter protein, is the strongest genetic risk factor for AD, with inheritance of the e4 variant associated with a substantially increased risk of AD (Harold et al., 2009; Lambert et al., 2009). Although a link between APOE and β-amyloid (Aβ) pathology is well established, experimental evidence also suggests that APOE exacerbates tau pathology independent of Aβ (Liu et al., 2013; Rebeck et al., 1993; Shi et al., 2017; Therriault et al., 2020).

Niemann-Pick type C (NPC) is a rare autosomal recessive disorder characterized by aberrant accumulation of intracellular cholesterol and glycolipids. Approximately 95% of NPC cases are caused by loss-of-function mutations in the Niemann-Pick C1 gene (*NPC1*), which encodes a broadly expressed trafficking protein important for transport of cholesterol to organelles and the plasma membrane from the lysosome (Garver et al., 2002; Vanier et al., 1996). NPC is a progressive childhood neurological disease, often with abundant tau pathology (Love et al., 1995). Thus, cholesterol abundance and localization are strongly associated with tau pathology, but a mechanistic link between cholesterol and tau pathology remains obscure.

In this study, we develop highly sensitive methods for detection of tau entry into the cytosol, permitting analysis of entry at physiological concentrations of tau. We find that entry of tau into the cytosol is the rate-limiting step in seeded aggregation. We observe that membrane cholesterol is a critical determinant of tau entry into neurons. Depletion of cholesterol from the plasma membrane, or its mislocalization after NPC1 depletion, promoted tau entry. This increased entry augmented seeded aggregation in organotypic slice culture and human and mouse neurons. These results establish cytosolic entry as an event distinct from uptake that is essential for the seeded aggregation of tau. They reveal cholesterol to be a critical determinant of tau entry into the cell interior, providing mechanistic insights into the relationship between cholesterol and tau pathology.

## Results

### Recombinant tau-HiBiT assemblies can reconstitute NanoLuc *in vitro* and in cells

**Figure 1:**
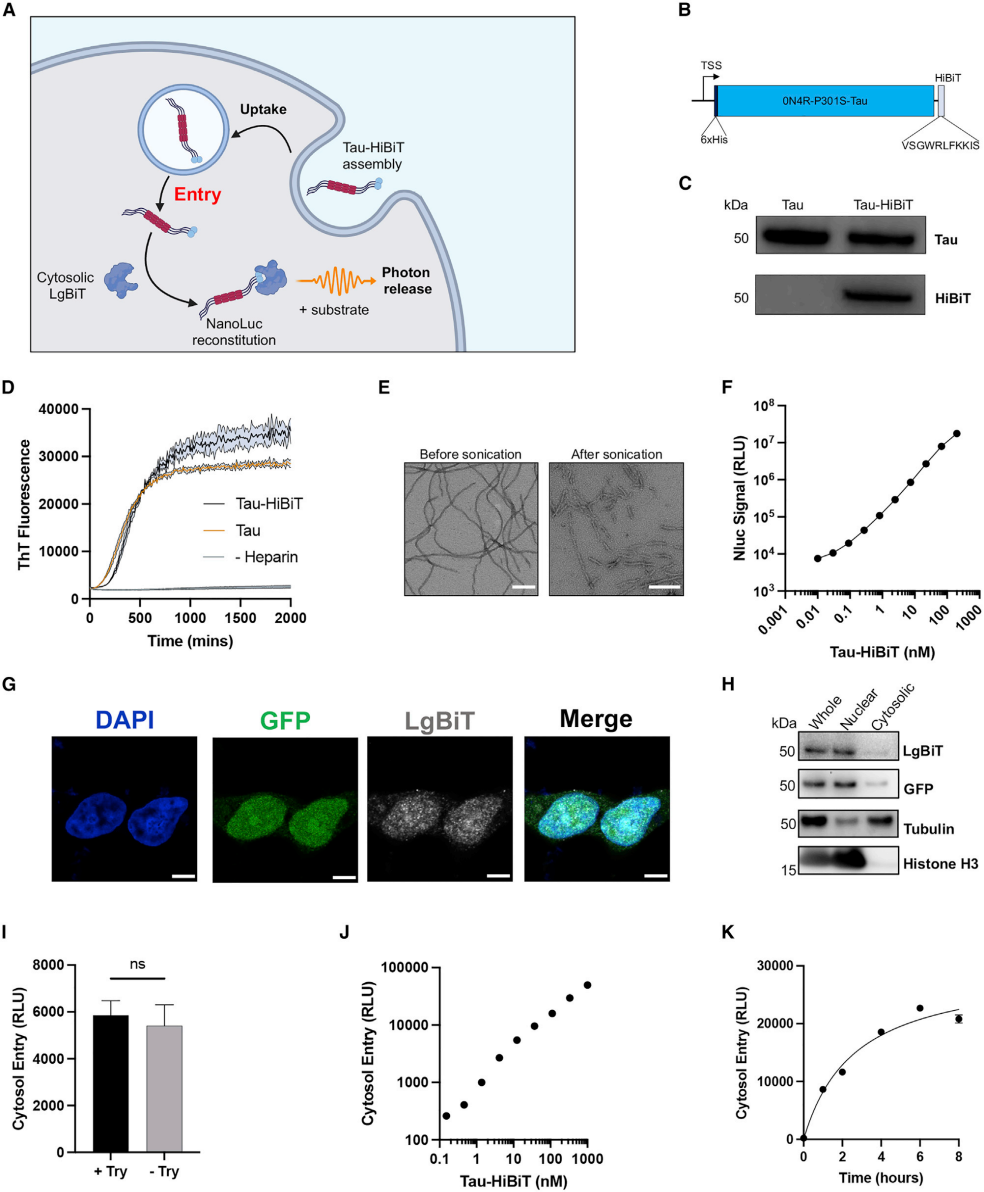
Characterization of HiBiT-tagged tau assemblies and their entry into the cytosol of HEK293 cells (A) Cartoon depicting the intracellular reconstitution of Nluc and the enzymatic production of light through interaction of exogenously supplied assemblies of tau-HiBiT with intracellular LgBiT. (B) Depiction of the His_6_-0N4R-P301S-Tau-HiBiT construct and the amino acid sequence of the HiBiT peptide. (C) Western blot of 50 ng recombinant tau or tau-HiBiT monomers with anti-tau (Dako) or anti-HiBiT antibody. (D) Time course of 5 μM tau-HiBiT and tagless tau aggregation kinetics monitored by thioflavin T (15 μM) fluorescence; n = 4. (E) Representative transmission electron micrographs of heparin-induced tau-HiBiT assemblies before and after sonication. Scale bar, 200 nm. (F) Titration of tau-HiBiT assemblies complexed with recombinant LgBiT (0.2 μL/well) *in vitro* for 30 min; n = 4. (G) Confocal microscopy images of HEK293T cells expressing NLS-EGFP-LgBiT (HEK-NGL), immunostained with anti-GFP and anti-LgBiT antibodies. Scale bars, 50 μm. (H) Western blot of cytosolic and nuclear fractions of NGL lysates probing for LgBiT, GFP, the nuclear marker histone H3, and the cytosolic marker tubulin. (I) Effect of trypsin protease (Try) treatment, which degrades extracellular luciferase, on the luminescent signal in NGL cells; n ≥ 3. (J) Titration of tau-HiBiT assemblies on NGL cells for 1 h; n = 3. (K) Time course of entry of 50 nM tau-HiBiT assemblies added to NGL cells; n = 3. All error bars indicate mean ± SEM.

Study of tau entry to cells has been complicated by the difficulty of reliably distinguishing cytosolic populations from vesicular populations (De La-Rocque et al., 2021). To specifically detect the cytosolic fraction of exogenously supplied tau assemblies, we established a live-cell assay relying on the split luciferase NanoLuc binary technology system (NanoBiT) (Figure 1A). The NanoBiT system relies on the NanoLuc (Nluc) enzyme, which is a 19-kDa luminescent protein engineered from the luciferase of the deep-sea shrimp *Oplophorus gracilirostris.* This enzyme has been split into a larger 18-kDa subunit (LgBiT) and an 11-amino-acid high-affinity peptide (HiBiT) that interact with subnanomolar affinity. Reconstitution results in complementation of activity and luminescence in the presence of substrate (Dixon et al., 2016). Previous studies have demonstrated the applicability of split luciferase as a tool to monitor tau aggregation and propagation mechanisms (Mirbaha et al., 2015; Wegmann et al., 2016). We expressed recombinant P301S tau (0N4R isoform) in fusion with a HiBiT tag at the C terminus in *Escherichia coli* (Figures 1B and 1C)*.* Assemblies of tau-HiBiT were produced by incubation with heparin and aggregation kinetics were quantified by thioflavin T fluorescence. Tau-HiBiT assembled into filaments, as seen by electron microscopy, and had similar aggregation profiles as tagless tau (Figures 1D and 1E).

To assess the ability of tau-HiBiT assemblies to reconstitute Nluc, we titrated tau-HiBiT assemblies into a fixed concentration of recombinant LgBiT, resulting in a concentration-dependent increase in luminescent signal (Figure 1F). We found that the HiBiT tag was as readily accessible in assemblies as it was in a monomer because the signal was unchanged between these states (Figure S1).

To measure entry of tau assemblies into the cell, we expressed LgBiT in the cytosol of HEK293 cells, a cell line widely used in the study of seeded tau aggregation (Falcon et al., 2014; Frost et al., 2009; McEwan et al., 2017; Woerman et al., 2016). We expressed LgBiT by lentivirus from the vector pSMPP (Clift et al., 2017), which drives expression from a spleen focus-forming virus (SFFV) promoter. We then applied sonicated tau-HiBiT assemblies to the cell exterior. We observed a bioluminescent signal after addition of cell-penetrant Nluc substrate. However, this signal was sensitive to addition of trypsin, a protease that degrades cell-free tau-HiBiT:LgBiT complexes (Figure S1). We interpreted this as an indication that the bulk of the interaction was extracellular, likely because of leakage or secretion of LgBiT into the surrounding medium.

We hypothesized that reducing the cytosolic concentration of LgBiT might be key to obtaining a signal that was exclusively intracellular. This was performed by use of a low-activity phosphoglycerate kinase (PGK) promoter and addition of a nuclear localization signal (NLS) to sequester much of the construct in the nucleus, relying on nucleocytoplasmic shuttling to maintain a small pool of LgBiT in the cytosol. The subnanomolar affinity of HiBiT and LgBiT was expected to be sufficient to drive luciferase reconstitution in the small fraction of tau-HiBiT assemblies that reach the cytosol. The resulting construct (NLS-EGFP-LgBiT [NGL]) exhibited an ∼85:15 nuclear:cytoplasmic ratio. Here, the luciferase signal was not quenched by trypsin after treatment of cells with tau-HiBiT (Figures 1G–1I). We next titrated tau-HiBiT onto HEK293 cells expressing the construct (HEK-NGL) and observed a dose- and time-dependent signal that saturated around 6 h (Figures 1J and 1K). These results demonstrate that the assay reports real-time entry of tau into live cells with a broad and linear dynamic range. To determine whether LgBiT concentration was also important to the bioluminescent signal, we prepared cell lines expressing high or low levels of LgBiT (Figure S1). We found that the luminescent signal was independent of the intracellular LgBiT concentration. This contrasts tau-HiBiT, where a linear relationship between concentration and the bioluminescence signal was observed. Bioluminescence can therefore be used in an entry assay to determine the levels of tau-HiBiT in the intracellular domain.

### Tau-HiBiT assemblies enter cell lines via clathrin-mediated endocytosis

**Figure 2:**
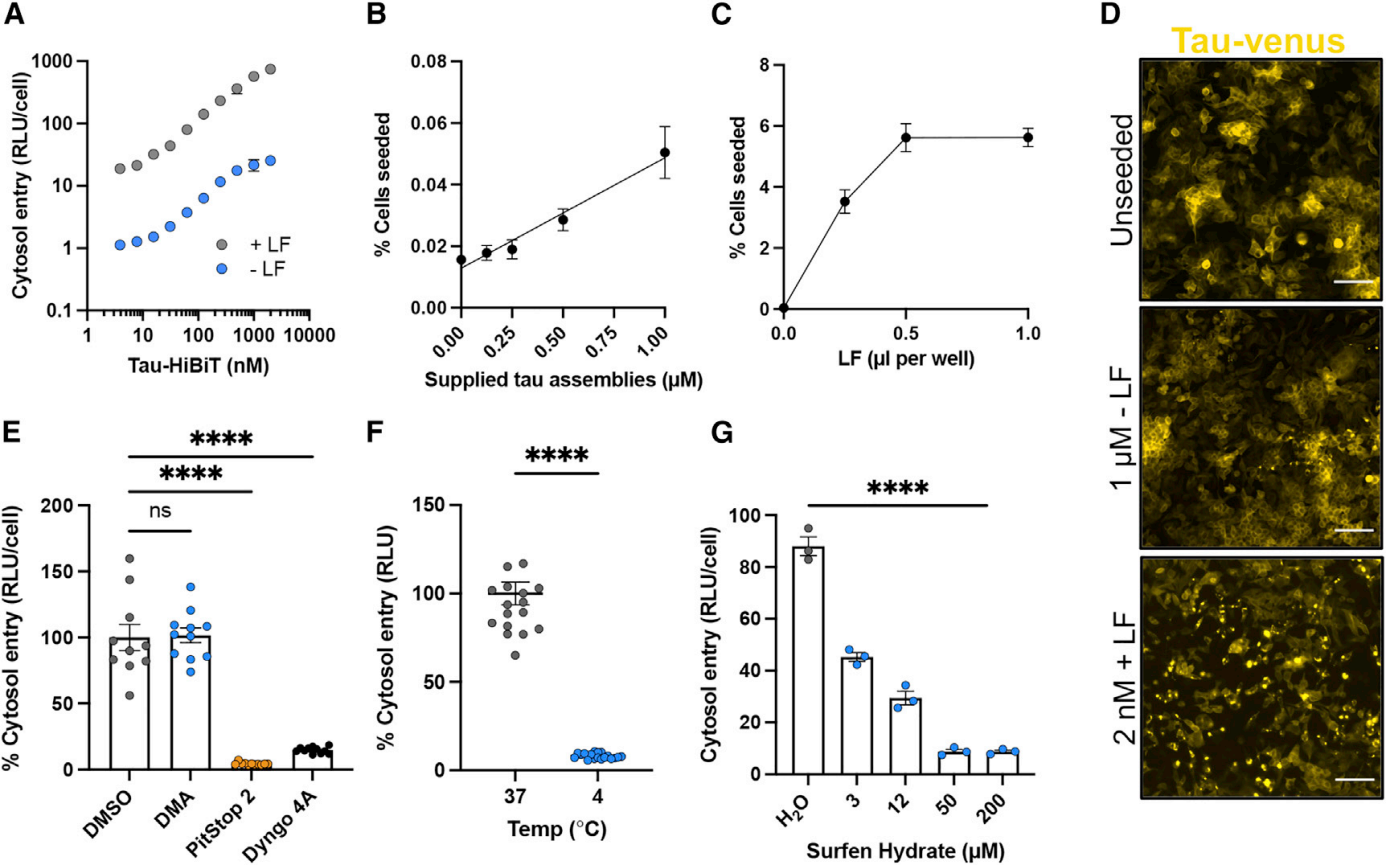
Entry of tau assemblies into HEK293 cells relies on clathrin- and dynamin-dependent endocytosis (A) Titrations of tau-HiBiT assemblies with or without Lipofectamine 2000 (LF) onto NGL cells. Entry was measured 24 h after challenge; n = 3. (B) Percentage of P301S tau-venus cells seeded 72 h after challenge with the indicated concentration of tau assemblies; n = 3. (C) Percentage of P301S tau-venus cells seeded 72 h after challenge with 2 nM tau assemblies supplemented with the indicated concentrations of LF; n = 3. (D) Fluorescence microscopy images of unseeded control (PBS-treated) P301S tau-venus cells, cells challenged with 1 μM tau assemblies without LF, or cells challenged with 2 nM tau assemblies with LF. Scale bars, 100 μm. (E) Effect of uptake inhibitors on entry of 50 nM of tau-HiBiT assemblies on HEK-NGL cells 1 h after challenge. HEK-NGL cells were pre-treated with DMA (200 μM), PitStop 2 (20 μM), Dyngo 4a (20 μM), or solvent (DMSO) for 30 min before challenge; n ≥ 3, N = 3 independent experiments. (F) Effect of temperature on tau entry. 50 nM tau-HiBiT assemblies were supplied to HEK-NGL cells for 1 h at 37°C or 4°C; n = 16. (G) The effect of surfen hydrate on tau entry to HEK-NGL cells in 1 h. Cells were pre-treated for 30 min with the indicated concentrations or equivalent dilutions of solvent (water) before 50 nM tau-HiBiT addition; n = 3. All error bars indicate mean ± SEM. ∗∗∗∗p < 0.0001 by one-way ANOVA with Tukey's comparisons (E and G) or Student's t test (F).

Tau seeding can be increased by use of transfection reagents (Holmes et al., 2013), likely because of enhanced delivery of tau assemblies to the cell interior. To test whether use of transfection reagents result in increased cytosolic penetration of tau, we titrated tau-HiBiT assemblies in the presence and absence of Lipofectamine 2000 (LF) onto HEK-NGL cells. We observed a 50- to 100-fold increase in entry of tau to the cytosol when supplemented with LF (Figure 2A). In HEK293 cells expressing P301S tau-venus (McEwan et al., 2017), we observed an attendant increase in seeded aggregation in the presence of LF (Figures 2B–2D). These data demonstrate that LF promotes entry into the cell and implicates breaching of intracellular membranes as rate-limiting to seeded aggregation.

Tau uptake occurs via endocytosis and relies on the scaffold clathrin and the GTPase dynamin (Calafate et al., 2016; Evans et al., 2018, 2020; Shrivastava et al., 2019). We pre-treated cells with the clathrin inhibitor PitStop 2 or the dynamin inhibitor Dyngo 4a and performed a 1-h tau entry assay. Both inhibitors resulted in a stark reduction in tau entry into the cytosol (Figure 2E). We also treated cells with dimethyl-amiloride (DMA), which inhibits macropinocytosis (von Delwig et al., 2006; Grinstein et al., 1989), but observed no change in entry of tau-HiBiT assemblies into HEK-NGL cells (Figure 2E). Incubation of cells at 4°C, a temperature non-permissive to endocytosis, also prevented tau entry into the cells (Figure 2F). We confirmed the activity of these inhibitors in preventing the uptake of fluorescently labeled transferrin and in not affecting the nuclear:cytoplasmic ratio of NLS-GFP-LgBiT (Figure S2). HSPGs mediate uptake of tau into intracellular compartments and promote seeded aggregation (Holmes et al., 2013; Rauch et al., 2018). Pre-treatment with surfen hydrate, an agent that binds heparan sulfate, strongly diminished entry in our assay (Figure 2G), as did addition of exogenous heparin to the cell medium (Figure S2). To further validate these findings, we used confocal microscopy to visualize uptake/entry of GFP-labeled tau assemblies. We found that treatments thatprevent uptake of tau-HiBiT assemblies also reduced the number of tau-GFP puncta in the cell (Figure S2). These data confirm that tau assemblies are rapidly taken up into HEK293 cells via a process that relies on clathrin, dynamin, and HSPGs. Subsequent entry into the cytosol is necessary for seeded aggregation.

We next investigated whether the entry pathway was conserved between monomeric and assembled tau. We found that monomeric tau-HiBiT was insensitive to clathrin and dynamin inhibition but, in contrast to assemblies, was somewhat sensitive to macropinocytosis inhibition (Figure S2). These results suggest that assembled and monomeric tau may enter the cytosol by overlapping but distinct pathways, consistent with previous findings (Evans et al., 2018). As expected, however, monomeric tau species were unable to induce seeded aggregation (Figure S2).

### Compromised endolysosomal machinery increases tau entry

**Figure 3:**
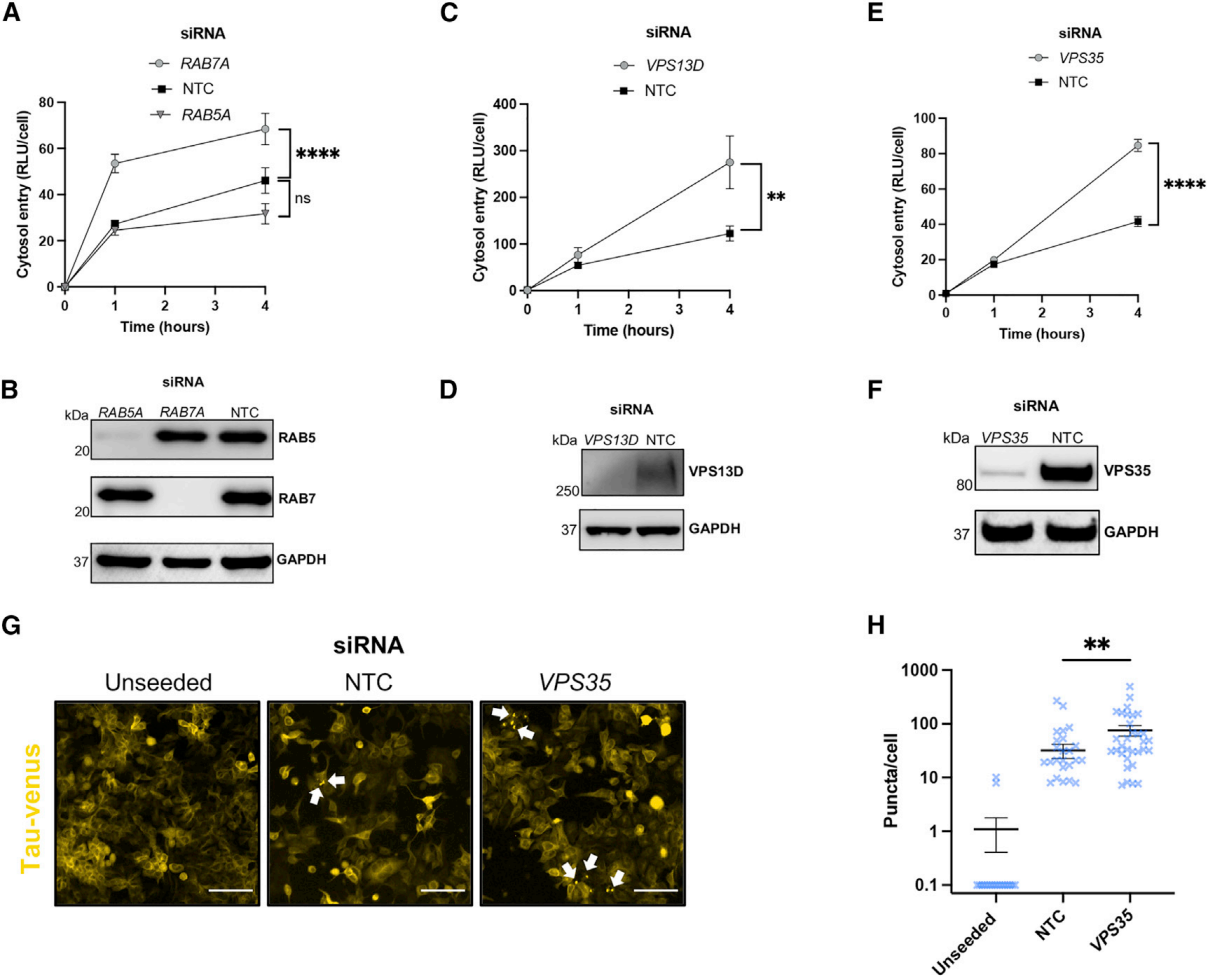
Defective endocytic machinery permits tau access to the cytosol in HEK-NGL cells (A) Levels of entry of 50 nM tau-HiBiT assemblies applied to HEK-NGL cells after RAB5A or RAB7A depletion. Cells were treated with siRNA or non-targeting control (NTC) siRNA for 72 h before assaying tau entry; n = 3, N = 3 independent experiments. (B) Western blot of cell lysates in (A), probing for RAB5, RAB7, and GAPDH. (C–F) Entry of tau-HiBiT assemblies into HEK-NGL cells after depletion of VPS13D (C and D) or VPS35 (E and F) and confirmation via western blotting following the same experimental procedure described in (A) and (B). (G) Fluorescence microscopy images of seeded tau-venus cells treated with NTC siRNA or *VPS35*-targeting siRNA for 72 h before addition of 250 nM exogenous tau assemblies in the absence of transfection reagents for another 72 h. Scale bars, 30 μm. White arrows indicate example fluorescent puncta. (H) Quantification of seeded aggregation from the tau-venus seeding assays in (G); n = 6. All error bars indicate mean ± SEM. ∗∗p < 0.01 by one-way ANOVA with Tukey's comparisons (C) or Kruskal-Wallis test with Dunn's comparisons (H). ∗∗∗∗p < 0.0001 by one-way ANOVA with Tukey's comparisons (A and E).

Clathrin- and dynamin-dependent endocytosis results in enclosure of cargo in a primary endocytic vesicle that undergoes maturation with involvement of Ras-associated binding (Rab) GTPases. To investigate the nature of the compartment from which tau escapes in HEK293 cells, we used small interfering RNA (siRNA) against early endosomal *RAB5A* or late endosomal *RAB7A* and performed tau entry assays over the course of 4 h (Figures 3A and 3B). Interestingly, we observed an increase in entry when RAB7 was depleted but not RAB5, suggesting a sensitivity of entry to impaired late endosomal compartments.

It has been shown that compromised function of VPS13D, a membrane bridging protein with ubiquitin- and lipid-binding capacity (Guillén-Samander et al., 2021; Wang et al., 2021), promoted seeded aggregation of tau (Chen et al., 2019). It has been proposed that the potential mechanism of increased tau aggregation may be a result of enhanced endolysosomal escape of tau seeds. To determine whether VPS13D is involved in preventing tau assemblies from accessing the cytosol, we depleted VSP13D in HEK-NGL cells and observed a marked increase in tau entry (Figures 3C and 3D). This corroborates the finding that VPS13D plays a role in maintaining tau from entering the cytosol.

We next investigated the role of VPS35, a core constituent of the endosome-to-Golgi apparatus retromer complex. Deficiencies in *VPS35* are linked to an increased tau burden and late-onset AD (Vagnozzi et al., 2019; Wen et al., 2011) and are a known genetic risk in Parkinson’s disease (Vilariño-Güell et al., 2011). We observed a significant increase in tau entry in cells depleted of VPS35 (Figures 3E and 3F). To support these findings, we performed a 1-h tau-GFP uptake assay in HEK293T cells followed by confocal microscopy. A proportion of tau-GFP assemblies were localized with VPS35, the early endosomal marker EEA1, and the late endosomal marker RAB7. These findings confirm a relationship between tau assemblies and the endosomal network (Figure S3). We next tested whether VPS35 was required to prevent seeded aggregation and observed a significant increase in seeding after its depletion (Figures 3G and 3H). These data demonstrate an essential role of endosome sorting and repair machinery in preventing tau escape to the cytosol in HEK293 cells.

### The mechanism of tau entry is cell type dependent

**Figure 4:**
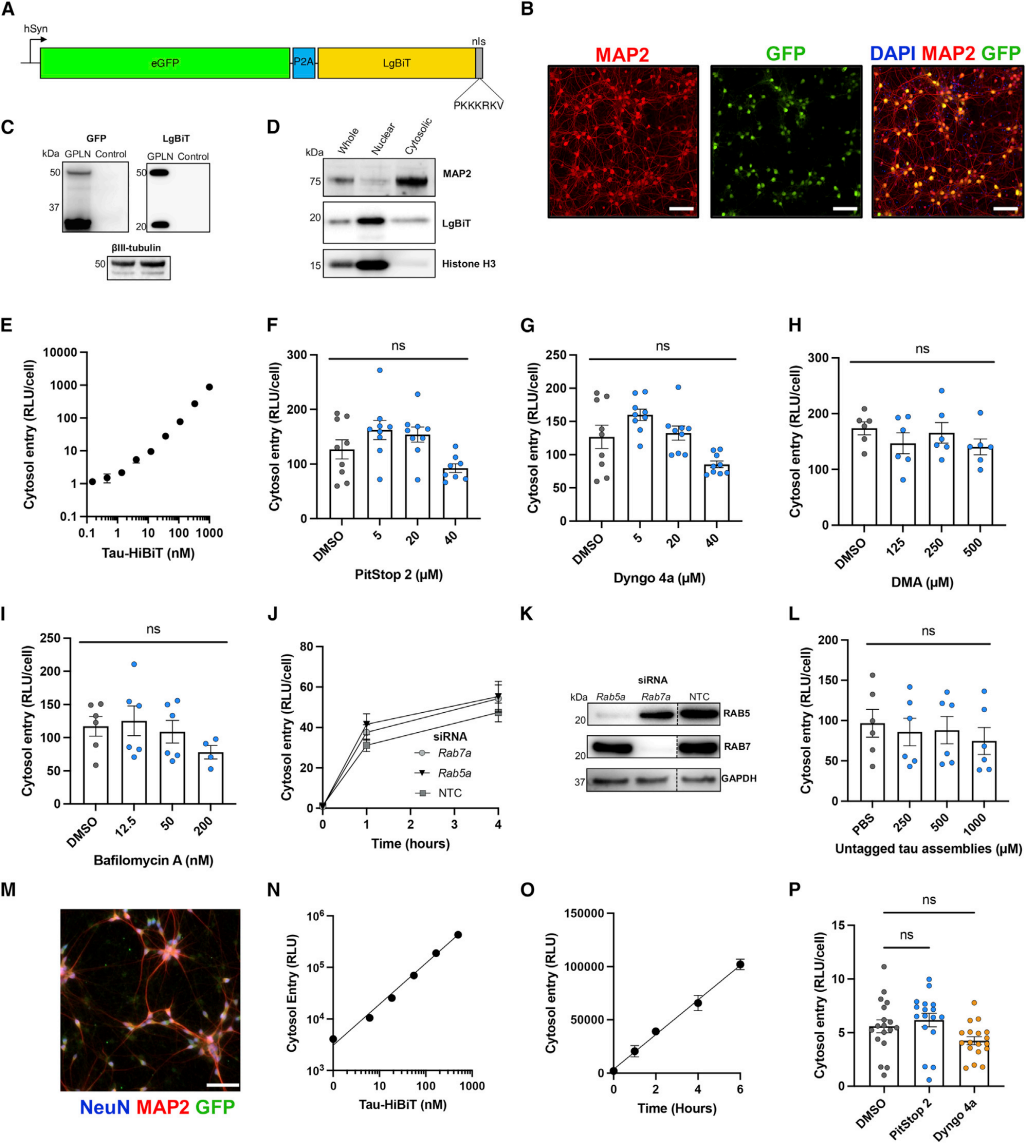
Entry into mouse and human neurons is clathrin and dynamin independent and not mediated by tau assemblies themselves (A) Cartoon depicting the GFP-P2A-LgBiT-NLS construct that was introduced to neurons by AAV1/2 under a human synapsin (hSyn) promoter. (B) Fluorescence microscopy images of DIV 7 WT neurons transduced with 50,000 genome copies (gc)/cell AAV1/2-hSyn-GPLN on DIV 2 (GPLN neurons). Scale bars, 100 μm. (C) Western blot of GPLN-neurons shown in (B), probing for GFP, LgBiT, and βIII-tubulin. (D) Western blot of whole-cell, nuclear, and cytosolic fractions of GPLN neurons, probing for LgBiT. MAP2 was used as a cytosolic control and histone H3 as a nuclear control. (E) Entry of tau-HiBiT assemblies 1 h after addition to GPLN neurons; n = 3. (F–I) Effect of clathrin (F), dynamin (G), macropinocytosis (H), or vacuolar ATPase (I) inhibition on entry of 50 nM tau-HiBiT assemblies into GPLN-neurons. GPLN neurons were pre-treated for 1 h with the indicated compound or solvent control. n ≥ 3, N = 3 independent experiments (F and G); n = 3, N = 2 (H and I). (J and K) Entry of 50 nM tau-HiBiT assemblies in GPLN neurons 72 h after knockdown with *Rab5a*, *Rab7a*, or NTC siRNA (J); n = 3, N = 3 independent experiments. Protein depletion was confirmed via western blot (K). For the full blot, see Figure S4. (L) Entry assay of 50 nM tau-HiBiT assemblies in the presence of increasing concentrations of untagged tau assemblies or solvent (PBS) as a control. Untagged tau assemblies were co-incubated with tau-HiBiT assemblies, and entry was quantified after 1 h; n = 3, N = 2 independent experiments. (M) Fluorescence microscopy images of day 14 iNeurons transduced with a total of 200,000 gc/cell AAV1/2-hSyn-GPLN. (N) Titration of tau-HiBiT assemblies added to GPLN-expressing iNeurons, with entry assayed after 1 h; n = 3. (O) Entry of 50 nM tau-HiBiT assemblies into GPLN-expressing iNeurons, with entry assayed at the indicated time; n = 3. (P) Effect of clathrin and dynamin inhibition on tau entry into GPLN-expressing iNeurons. Neurons were pre-treated with PitStop 2 (30 μM), Dyngo 4a (30 μM), or solvent (DMSO) for 1 h prior to challenge with 50 nM tau-HiBiT assemblies. Entry was measured at 1 h. n = 3–6 from N = 3 independent differentiations. All error bars indicate mean ± SEM.

To investigate the mechanism of tau entry in a more physiologically relevant system, we adapted our assay to primary neurons derived from wild-type (WT) C57BL/6 mice. We used chimeric particles of an adeno-associated virus with capsids of serotypes 1 and 2 (AAV1/2) to deliver a self-cleaving variant of the LgBiT reporter from a neuron-specific synapsin promoter (hSyn). This construct, hSyn::-EGFP-P2A-LgBiT-NLS (GPLN), provides EGFP as a fluorescent marker and a 70:30 nuclear:cytosolic ratio of LgBiT with close to 100% transduction efficiency (Figures 4A–4D and S4).

Challenge of GPLN neurons with tau-HiBiT generated a dose-responsive signal that was resistant to trypsin digestion, confirming its intracellular origin (Figures 4E and S4). To determine the uptake pathway that results in tau entry to the cytosol, we treated neurons with inhibitors of clathrin and dynamin. Surprisingly, neither inhibitor reduced entry of tau into the cytosol (Figures 4F and 4G) despite successfully reducing transferrin uptake (Figure S4). Given that tau can spread in a transsynaptic manner (Calafate et al., 2015; Liu et al., 2012; Wu et al., 2016), we considered whether the observed independence was a result of performing entry assays in cells with improperly formed synapses, which occurs around 7–10 days *in vitro* (DIV) (Grabrucker et al., 2009; Verstraelen et al., 2018). We therefore performed entry assays in DIV 14 neurons but consistently found no role of clathrin or dynamin in entry of tau assemblies or monomers into the cytosol of neurons (Figure S4). Similarly, treatment with DMA was unable to prevent entry of tau assemblies (Figure 4H) or monomers (Figure S4).

We next treated neurons acutely (1 h) with the vacuolar-type ATPase (V-ATPase) inhibitor bafilomycin-A to inhibit endosome acidification but not autophagy (Yoshimori et al., 1991). We observed no significant effect on tau entry (Figure 4I). Neuronal viability was monitored to ensure that experiments reflected entry into healthy cells (Figure S4). We next depleted mouse RAB5 or RAB7 via siRNA and monitored tau-HiBiT entry over 4 h (Figures 4J and 4K). Despite a knockdown efficiency of more than 90% and minimal toxicity (Figure S4), we found no role of either protein in tau entry into neurons, in direct contrast to HEK293 cells.

### Tau assemblies do not mediate their own escape to the cytosol

One possibility to explain the pathway independence is that tau assemblies mediate their own entry into the cytosol by actively destabilizing cell-limiting membranes. To test this, we titrated tagless tau assemblies in the presence of a constant (50 nM) concentration of tau-HiBiT. If the tagless tau assemblies promoted membrane rupturing, then we predicted this would result in an increase in tau-HiBiT entry. However, in HEK293 cells and primary neurons, we found no increase in signal, suggesting that tau does not mediate its own entry (Figures 4L and S5). We used a second membrane integrity assay where a plasmid encoding luciferase is supplied to the extracellular medium of HEK293 cells. Addition of membrane-rupturing agents such as an adenovirus or LF resulted in plasmid transfer to the intracellular environment, promoting luciferase expression. In contrast, tau assemblies and monomers resulted in no signal above background (Figure S5). We found no evidence that tau escapes to the cytosol by active disruption of cell-limiting membranes.

### Tau entry into human neurons is independent of clathrin and dynamin

To investigate entry of tau into human neurons, we differentiated induced pluripotent stem cells by expression of the NGN2 transcription factor (Pawlowski et al., 2017) and transduced them with AAV1/2-hSyn-GPLN (GPLN-iNeurons) (Figures 4M and S5). On day 14, iNeurons expressed essential neuronal and synaptic genes indicative of fully differentiated human cortical neurons (Figure S5). We found that entry of tau assemblies occurred in a concentration- and time-dependent manner, and using endocytosis inhibitors as above, we found no role of clathrin or dynamin in tau entry (Figures 4N–4P and S5). Thus, entry to neurons occurs via a similar pathway in human and mouse neurons.

### Tau entry is dependent on LRP1 and HSPGs

**Figure 5:**
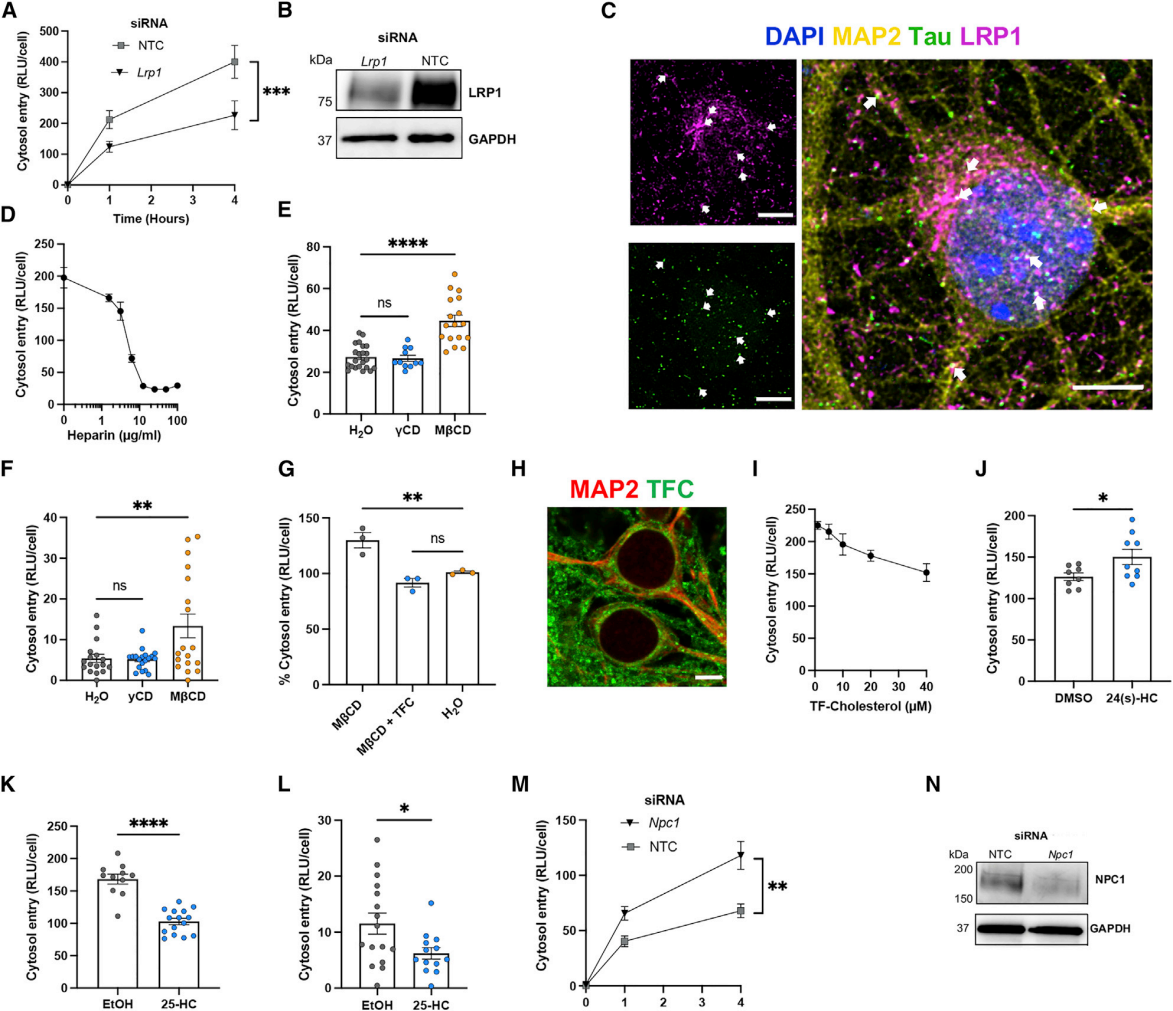
Entry of tau assemblies into mouse primary neurons depends on receptor-mediated uptake and is controlled by cholesterol (A) Entry of 50 nM tau-HiBiT assemblies into GPLN neurons in 1 h, 72 h after knockdown with *Lrp1* or NTC siRNA; n = 3, N = 3 independent experiments. (B) Western blot for LRP1 and GAPDH in cells from (A). (C) Confocal microscopy images (z stack) of WT neurons (DIV 7) immunostained for LRP1 and MAP2 after a 1-h tau uptake assay with 200 nM tau-GFP assemblies. White arrows indicate examples of colocalization of tau assemblies and LRP1. Scale bar, 5 μm. (D) Entry of 50 nM tau-HiBiT assemblies into GPLN-neurons in the presence of the indicated concentration of heparin, with entry measured 1 h after challenge; n = 3. (E and F) Effect of cholesterol depletion in GPLN neurons (E) and GPLN-expressing human iNeurons (F). Neurons were pre-treated with γCD (2 mM), MβCD (2 mM), or solvent (water) for 2 h before addition of 50 nM tau-HiBiT assemblies for 1 h; n ≥ 4, N = 3 independent experiments/differentiations (E and F). (G) Effect of exogenous cholesterol on tau entry in cholesterol-depleted GPLN neurons. Cells were pre-treated with MβCD (500 μM) or solvent (water) with or without 10 μM TopFluor-cholesterol (TF-cholesterol; TFC) for 2 h before challenge with 50 nM tau-HiBiT assemblies. Entry was measured 1 h after challenge with tau-HiBiT assemblies; n = 3. (H) Confocal microscopy image of WT DIV 7 primary neurons with 10 μM TF-cholesterol added 16 h before. Scale bar, 5 μm. (I) Titration of TF-cholesterol onto GPLN neurons for 1 h prior to a 1-h entry assay with 50 nM tau-HiBiT assemblies; n = 3. Pearson correlation coefficient (r)= −0.98, ^∗∗^p = 0.0041. (J and K) Entry of 50 nM tau-HiBiT assemblies in GPLN neurons 1 h after challenge, which followed 1-h pre-treatment with 10 μM 24(s)-HC (J), 25-HC (K), or solvent (DMSO or ethanol [EtOH]); n = 3, N = 3–4 independent experiments. (L) Entry of 50 nM tau-HiBiT assemblies into GPLN-expressing iNeurons in 1 h after 1-h pre-treatment with 10 μM 25-HC or solvent (EtOH); n = 3, N = 3 independent differentiations. (M and N) Entry of 50 nM tau-HiBiT assemblies into GPLN neurons 1 h after 72-h treatments with siRNA against *Npc1* or NTC siRNA (M) with western blot to confirm protein depletion (N); n = 3, N = 3 independent experiments. All error bars indicate mean ± SEM. ∗p < 0.05 by Student's t test (L and J), ∗∗p < 0.01, ∗∗∗p < 0.001, ∗∗∗∗p < 0.0001 by one-way ANOVA with Tukey's comparisons (A, E, F, G, K, and M).

We next investigated the receptor dependency of tau entry. We depleted the tau uptake receptor LRP1 and observed substantially decreased tau entry into primary mouse neurons (Figures 5A and 5B). Consistent with this, we observed colocalization of LRP1 and tau-GFP assemblies by confocal microscopy (Figure 5C). Treatment of mouse and human neurons with heparin also reduced entry in a dose-dependent manner (Figures 5D and S6). Tau entry into neurons therefore depends on association with LRP1 and HSPGs.

### Tau entry is sensitive to cholesterol

We hypothesized that changes in membrane cholesterol may modulate entry of tau into the cytosol. To test this, we treated primary neurons with the cholesterol-extracting agent methyl-beta-cyclodextrin (MβCD) (Ilangumaran and Hoessli, 1998). Treatment with 2 mM MβCD for 2 h before addition of 50 nM tau-HiBiT for 1 h effectively extracted cholesterol from neuron membranes, as visualized by filipin staining, without cytotoxicity (Figure S6). Extraction resulted in a significant increase in tau entry into neurons (Figure 5E). Treatment with the control compound gamma-cyclodextrin (γCD), which does not extract cholesterol, resulted in no significant change in entry into human or mouse neurons (Figures 5E and 5F). Addition of the exogenous fluorescent cholesterol analog TopFluor cholesterol (TF-cholesterol) to cholesterol-depleted cells restored tau entry to levels observed in untreated cells (Figure 5G). To examine the effect of increasing membrane cholesterol, we pre-treated mouse neurons with increasing concentrations of TF-cholesterol. We found a dose-dependent reduction in entry when TF-cholesterol was supplied, suggesting that cholesterol acts as a barrier to entry (Figures 5H and 5I).

We were concerned that our observations were a result of increased extracellular signal because of LgBiT leakage when treated with MβCD. We therefore depleted cholesterol from neuronal membranes and performed a tau entry assay in the presence of the extracellular protease trypsin. We found no significant reduction in signal with trypsin, indicating that the signal originates in cells (Figure S6). We further tested whether removal of membrane cholesterol promoted non-specific loss of membrane integrity, permitting entry of other proteins. For this, we used free HiBiT peptide or GFP protein fused to HiBiT. We found that MβCD treatment resulted in no change in entry for either protein, suggesting that the effects of MβCD are specific to tau and not due to general membrane leakiness (Figure S6).

### Oxysterols and NPC1 affect tau entry

Brain cholesterol is secreted to the periphery after its modification to 24(s)-hydroxycholesterol (24(s)-HC) by the neuronal enzyme CYP46A1. In early dementia, 24(s)-HC is elevated in cerebrospinal fluid and correlates with tau pathology (Björkhem et al., 2018; Papassotiropoulos et al., 2002). We tested the effect of 24(s)-HC on tau entry in primary neurons by pre-treatment and a 1-h entry assay. Interestingly, we observed a significant increase in tau entry after treatment with 24(s)-HC (Figure 5J). Similarly, treatment with efavirenz, an anti-retroviral drug that activates CYP46A1 (van der Kant et al., 2019), increased entry of tau (Figure S6). A second naturally occurring cholesterol oxysterol, 25-hydroxycholesterol (25-HC), promotes accumulation of cholesterol in subcellular compartments (Liu et al., 2018) and has been shown to inhibit entry of viruses into the cell, including severe acute respiratory syndrome coronavirus 2 (SARS-CoV-2) (Gomes et al., 2018; Zang et al., 2020). We observed that treatment of neurons with 25-HC reduced tau entry in mouse and human neurons (Figures 5K and 5L). These data demonstrate that changes in membrane cholesterol and its hydroxy derivatives are important determinants of tau entry into the cytosol of neurons.

Mutations in the Niemann-Pick C1 gene (*NPC1*) cause NPC (Park et al., 2003), a disease of mis-sorted cholesterol that can feature pathological tau aggregation. We questioned whether depletion of NPC1 in primary neurons could modulate tau entry. We depleted NPC1 by siRNA and observed a significant increase in tau entry into primary neurons (Figures 5M and 5N). The role of NPC1 is to transport cholesterol to the plasma membrane, and loss of NPC1 in disease results in sequestration of cholesterol in subcellular compartments at the expense of the plasma membrane (Vance, 2012). Our results are therefore consistent with NPC1 depletion resulting in enhanced tau entry in a manner similar to cholesterol depletion by MβCD. These results suggest that intracellular cholesterol concentration and distribution are critical for entry of tau into the cytosol.

### Seeded aggregation of tau is highly sensitive to cholesterol dysregulation in neurons

To investigate the relationship between entry and seeded aggregation, we prepared DIV 7 neurons expressing human 0N4R P301S tau from P301S-tau transgenic mice (Allen et al., 2002). When challenged with exogenous tau assemblies, intracellular phosphorylated tau aggregates can be detected by immunostaining with the antibody AT8, which detects assemblies phosphorylated at Ser202/Thr205. We performed seeded aggregation assays with 100 nM tau assemblies in the presence or absence of PitStop 2 or Dyngo 4a for 7 days. We observed no change in the extent of AT8-positive tau inclusions 7 days after the challenge in cells treated with pathway inhibitors compared with solvent-treated control neurons (Figure S7), and monomeric tau failed to induce seeded aggregation in this system (Figure S7). This suggests that seed-competent tau species enter the cytosol of neurons via a similar pathway as tau-HiBiT assemblies.

**Figure 6:**
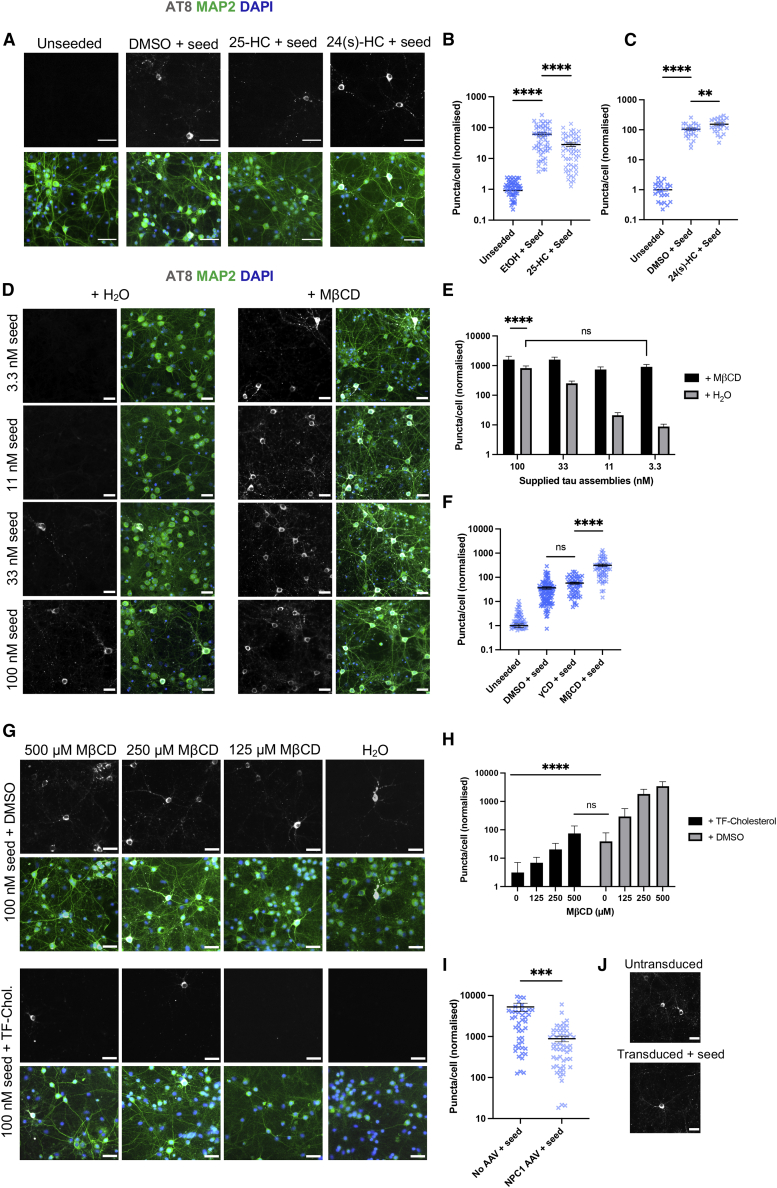
Cholesterol depletion promotes seeded aggregation of tau (A–C) Fluorescence microscopy images (A) and quantification of seeding assays in neurons prepared from P301S tau-transgenic mice after pre-treatment with 25-HC (10 μM), 24(s)-HC (10 μM), or solvent (DMSO) for 16 h prior to addition of 100 nM tau assemblies for 7 days. AT8-positive puncta were quantified (B and C). Scale bars, 30 μm. n = 3, N = 4 independent experiments (B), n = 3 (C). (D and E) Fluorescence microscopy images (D) and quantification of seeding assays with the indicated concentration of tau assemblies in the presence of MβCD or solvent control (water) in neurons prepared from P301S tau-transgenic mice. Neurons were pre-treated overnight with MβCD (500 μM) prior to a 7-day seeding assay with the depicted concentrations of tau assemblies. AT8-positive puncta were quantified (E). Scale bars, 30 μm. n = 3, N = 3 independent experiments. (F) Quantification of seeded aggregation in neurons prepared from P301S tau-transgenic mice 7 days after challenge with 100 nM tau assemblies after overnight pre-treatment with MβCD (500 μM), γCD (500 μM), or solvent (DMSO); n = 3, N = 3 independent experiments. (G and H) Fluorescence microscopy images (G) and quantification of seeded aggregation in neurons prepared from P301S tau-transgenic mice after pre-treatment with the depicted concentrations of MβCD plus 10 μM TF-cholesterol or solvent (DMSO) overnight prior to addition of 100 nM tau assemblies for 7 days. AT8-positive puncta were quantified (H). Scale bars, 30 μm. n = 3, N = 3 independent experiments. (I and J) Quantification (I) and fluorescence microscopy images (J) of seeded tau aggregation in neurons prepared from P301S tau-transgenic mice transduced with AAV1/2-hSyn-NPC1. Cells were transduced with 100,000 gc/cell on DIV 3, followed by a 7-day seeding assay on DIV 7. AT8-positive puncta were quantified (I). Scale bars, 30 μm. n = 3, N = 3 independent experiments. All error bars indicate mean ± SEM. ∗∗p < 0.01, ∗∗∗p < 0.001, ∗∗∗∗p < 0.0001 by Kruskal-Wallis test with Dunn's comparisons (B, C, and F), Student's t test (I), or two-way ANOVA with Sidak's comparisons (E and H).

We next probed whether the changes in tau entry observed after modulation of cholesterol were mirrored by changes in seeded aggregation. First we treated neurons with 25-HC or 24(s)-HC, which impaired and promoted entry, respectively. We observed significant changes in seeded aggregation when neurons were treated with these compounds, consistent with the effects on entry (Figures 6A–6C). We next explored the effect of cholesterol itself on seeded aggregation in neurons. We extracted cholesterol with a low, well-tolerated level of MβCD (500 μM) and challenged cells with a titration of tau assemblies (Figures 6D and 6E). In the absence of MβCD, we observed seeded aggregation after challenge with tau assemblies at or above 33 nM. Remarkably, in the presence of MβCD, substantial seeding was observed at 3 nM tau assemblies. Levels of seeding in cholesterol-depleted neurons challenged with 3 nM tau were comparable with levels observed with 100 nM tau assemblies in neurons that were not treated with MβCD. Importantly, we found no significant change in seeding with γCD- and solvent-treated controls (Figure 6F).

We next wanted to determine whether supplementing neurons with exogenous cholesterol could protect against seeded aggregation and reverse the effects of MβCD. Strikingly, we found that treatment with TF-cholesterol completely rescued the effect of MβCD and reduced seeded aggregation by more than 90% in neurons that had not been treated with MβCD (Figures 6G and 6H). The effect size of changing membrane cholesterol levels extended from virtually all cells containing aggregates to virtually no cells containing aggregates. This identifies membrane cholesterol as a key determinant of susceptibility to seeded aggregation and shows that the mechanism of cholesterol’s activity is preventing tau access to the cytosol.

Our entry data demonstrated that depletion of NPC1 promoted tau entry into neurons. We hypothesized that overexpression of NPC1 would impair entry and seeding by promoting cholesterol trafficking to the neuronal membrane (Millard et al., 2000). We used AAV1/2-hSyn-NPC1 to induce expression of human NPC1 in mouse neurons (Figure S7). We observed a small but significant reduction in entry at 1 h (Figure S7). We next wanted to determine whether this effect would be magnified in seeding assays. Here, we observed a substantial reduction in seeded aggregation when NPC1 was overexpressed (Figures 6I and 6J). These data suggest that the cholesterol transport function of NPC1 plays a critical role in preventing entry of tau assemblies into the cytosol, preventing seeded aggregation.

### Cholesterol depletion promotes widespread seeding in organotypic slice culture

**Figure 7:**
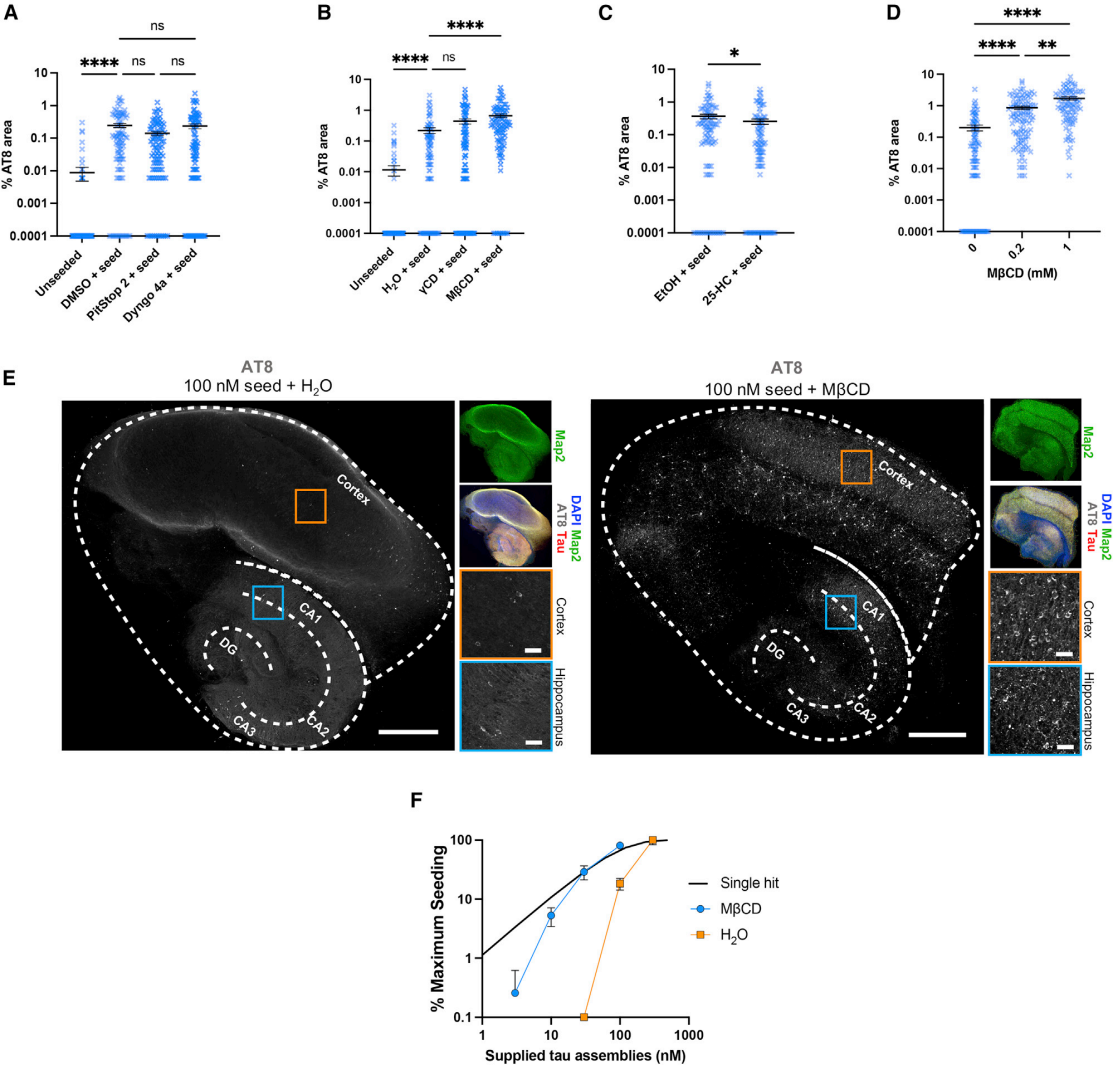
Cholesterol extraction promotes seeded aggregation in organotypic hippocampal slice cultures (A–C) Levels of seeded aggregation in brain slice cultures from P301S tau transgenic mice treated with the relevant solvent control or (A) endocytosis inhibitors (Pitstop 2, 10 μM; Dyngo 4a, 2 μM), (B) cyclodextrins (γCD and MβCD, 200 μM), and (C) 25-HC (10 μM) for 24 h prior to addition of fresh drug and 100 nM tau assemblies for 3 days. Medium was exchanged, and slice cultures were incubated for another 3 weeks prior to fixation and immunostaining. Slices are from N = 6 mice. (D) Quantification of seeded aggregation in slice cultures from P301S transgenic mice treated with 100 nM tau assemblies in the presence of 200 μM or 1 mM MβCD as in (A). Slices are from N = 6 mice. (E) Fluorescence microscopy images of entire slice cultures with details of hippocampal (blue) and cortical (orange) areas. OHSCs were seeded with 100 nM tau assemblies in the presence or absence of 1 mM MβCD. The cortex; hippocampal CA1, CA2, and CA3; and the dentate gyrus (DG) are labeled. Scale bar, 500 μm and 50 μm (hippocampus and cortex, respectively). (F) Percent maximum seeding in the presence of MβCD (200 μM) or solvent (water). Seeded aggregation was quantified in OHSCs from P301S tau transgenic mice. A black line indicates a single-hit curve. Slices are from N = 6 mice. All error bars indicate mean ± SEM. ∗p < 0.05 by Student's t test (C), ∗∗p < 0.01, ∗∗∗∗p < 0.0001 by Kruskal-Wallis test with Dunn's comparisons (A, B, and D)

We next sought to investigate whether changes in tau entry lead to changes in seeded aggregation in a physiological setting. We used organotypic hippocampal slice cultures (OHSCs), which maintain authentic neural architecture and cell type diversity (Croft et al., 2019). OHSCs prepared from P301S tau transgenic mice do not develop detectable tau pathology unless challenged with tau assemblies (Miller et al., 2021). Tau assemblies were provided to OHSCs with or without endocytosis inhibitors or cholesterol modulators, and AT8 staining was examined after 3 weeks (Figure S7). Inhibition of clathrin or dynamin resulted in no significant change in seeded aggregation (Figure 7A). Extraction of cholesterol with MβCD substantially increased seeded aggregation (Figure 7B), and treatment with 25-HC significantly reduced seeded aggregation (Figure 7C). We next treated OHSCs with 0.2 or 1 mM MβCD to remove membrane cholesterol and observed a dose-dependent increase in seeded aggregation in response to 100 nM tau assemblies (Figure 7D). Finally, we examined the spatial distribution of AT8-positive inclusions in hippocampal-cortical slices when cholesterol was extracted with MβCD. We observed a marked increase in AT8-positive inclusions throughout the hippocampus and cortex (Figure 7E). These findings confirm that cholesterol concentration is a critical determinant of seeded aggregation in neural tissue.

We have shown previously that the seeding activity of tau assemblies does not titrate in the single-hit manner that would be expected of independently acting particles in slice cultures (Miller et al., 2021). Instead, seeding was only observed when tau assemblies were supplied at 100 nM or above. Low concentrations of tau assemblies, as found in the extracellular spaces of neural tissue, failed to induce seeded aggregation. We hypothesized that cholesterol may contribute to the barrier against seeding at low tau concentration. We extracted cholesterol from OHSCs using 200 μM MβCD, or retained native cholesterol levels, and titrated tau assemblies. When MβCD was absent, seeding was only observed at high concentrations of tau assemblies, as shown previously (Miller et al., 2021). Following cholesterol extraction, however, seeding was observed even at low concentrations, and the response to dose approached single-hit dynamics (Figure 7F). These results suggest that low cholesterol potentiates seeded aggregation of tau at low seed concentration, a condition consistent with the prion-like propagation of tau.

## Discussion

In this study, we developed sensitive assays capable of detecting entry of tau into the cytosol after its application at nanomolar concentrations to the cell exterior. We described entry of tau into the cytosol in two cell-based models: HEK293 cells, which are widely used as reporters for seeded aggregation, and neurons, the major site of tau pathology in the brain of individuals with AD. Levels of entry in HEK293 and neurons were a critical determinant for seeded aggregation, consistent with entry being the rate-limiting step to seeded aggregation. In neurons, we found that entry was highly sensitive to levels of membrane cholesterol. Low cholesterol was associated with extreme permissivity to seeded aggregation by extracellular tau assemblies. The study presents tools for the study of tau biology and demonstrates a mechanistic link between cholesterol dysregulation and tau pathology.

### Cytosolic entry of tau is cell type dependent

We observed that the mechanism of entry was highly divergent between HEK293 cells and neurons. Entry in HEK293 cells is dependent on the vesicle coat protein clathrin and the GTPase dynamin, consistent with observations of tau uptake via canonical endocytosis in human immortalized cell lines (De La-Rocque et al., 2021). When we induced endolysosomal dysfunction in HEK293 cells via genetic knockdown of the late endosomal protein RAB7, we observed an increase in entry of tau assemblies. Similarly, knockdown of *VPS35*, which encodes a core constituent of the retromer complex and a RAB7 effector, also increased entry. We consider it likely that knockdown results in unstable vesicles that are liable to deliver their content to the cytosol. This is consistent with previous studies that have reported leaky vesicles upon induction of endolysosomal perturbation (Calafate et al., 2016; Nixon, 2017).

The dependency of canonical endocytic pathways and machinery was lost in primary mouse neurons and human iPSC-derived cortical neurons. Here, entry of tau assemblies occurred through a clathrin- and dynamin-independent pathway with no observable effect when Rab GTPases were depleted. This was not an artifact of using neurons with immature synaptic biology because DIV 14 mouse neurons and induced pluripotent stem cell (iPSC)-derived cortical neurons, which express markers of mature and functional neural networks, displayed a similar phenotype. Clathrin- and dynamin-independent endocytosis has been described for particular substrates, suggesting that tau entry occurs from a specific subset of endosomal compartments (Mayor et al., 2014) or potentially at the plasma membrane. Previous studies that have examined the uptake of tau to neurons have demonstrated involvement of clathrin-mediated endocytosis (Evans et al., 2018), macropinocytosis (Wu et al., 2013), and a clathrin-independent, dynamin-dependent, non-macropinocytic pathway (Soares et al., 2021). It is therefore possible that several pathways are responsible for tau uptake but that tau escape to the cytosol occurs from a restricted subset of compartments.

### Cholesterol and neurodegenerative disease

Cholesterol homeostasis has been repeatedly implicated in the risk and pathogenesis of AD (van der Kant et al., 2020). Polymorphisms in APOE, one of the principal transporters of lipids, including cholesterol, are the main genetic risk factors for AD. The e4 variant increases the genetic risk of AD in a dose-dependent manner. Research into the involvement of APOE in AD has focused predominantly on its effects on Aβ. However, recent data demonstrate that APOE polymorphisms also directly affect tau pathogenesis. APOE polymorphisms affect the risk of progressive supranuclear palsy, which does not feature Aβ pathology (Zhao et al., 2018), and APOE alleles modulate tau pathology independent of Aβ in animal models (Shi et al., 2019). The mechanism of APOE’s role in tau pathology remains unclear. Loss-of-function mutations in the intracellular cholesterol transporting protein NPC1 leads to Niemann-Pick disease, a severe form of early-onset neurodegeneration that features tau pathology. Heterozygous mice (*Npc1*^*+/−*^) have reduced levels of lipid raft cholesterol and demonstrate age-dependent tau pathology (Yu et al., 2005).

In the present study, we found that cholesterol levels in neurons are critical determinants of tau entry into the cytosol. Use of MβCD to extract cholesterol from membranes resulted in increased tau entry and high levels of seeding. This could be reversed by supplementing cultures with TF-cholesterol, which reduced tau entry and seeding. Genetic knockdown of *Npc1* leads to sequestration of cholesterol in intracellular compartments and, in our assay, an increase in tau entry. Conversely, a protective effect was observed when NPC1 was overexpressed in P301S primary neurons. Overexpression of this protein has been reported previously to enhance membrane insertion of cholesterol from subcellular compartments (Millard et al., 2000). The results are therefore consistent with cholesterol being critical for preventing tau assembly access to the cytosol. The magnitude of these effects was startling, with a 1,000-fold increase in seeding in neurons under conditions of low cholesterol (MβCD extracted) compared with high cholesterol (TF-cholesterol supplemented). In practical terms, this was the difference between the vast majority of neurons possessing AT8-positive aggregates and almost no AT8-positive aggregates being detected in response to a fixed concentration of extracellular tau assemblies.

The precise role of cholesterol in protection against tau entry remains uncertain. The cholesterol content of lipid membranes alters the physiochemical properties of lipid bilayers, increasing the rigidity and reducing the density of lipid packing (Dopico and Tigyi, 2007). Depletion of cholesterol using MβCD alters the mechanical parameters of membranes and can render them susceptible to rupture (Biswas et al., 2019). However, our results argue against a general degradation of membrane integrity when cholesterol was depleted because substrates other than tau (GFP-HiBiT and free HiBiT peptide) did not gain enhanced access to the cell interior after MβCD treatment. Potential mechanisms by which cholesterol may exert this protective effect are protection against failure of specific subcellular compartments or reduction of the passage of tau across the plasma membrane by altering the properties of the membrane or lipid rafts.

### Tau escape to the cytosol

It has been suggested that tau escape to the cytosol is dependent on membrane-destabilizing activities of tau itself (Calafate et al., 2015; Falcon et al., 2018; Flavin et al., 2017; Polanco and Götz, 2021). Tau assemblies can bind to cell membranes via interactions between the repeat region and phospholipid bilayers, potentially consistent with direct lysis of membranes (Ait-Bouziad et al., 2017). We found no evidence of tau-mediated entry, consistent with another recent study that found no entry-promoting characteristics of tau assemblies (Kolay et al., 2022). The different findings may reflect different cell types and fibril preparations or inherent differences when directly measuring endosomal lysis versus directly measuring tau cytosolic entry.

### Conclusion

We demonstrate that cytosolic entry is essential for and rate limiting to seeded aggregation of intracellular tau pools. Tau transit to the neuronal cytosol occurs after interactions with the receptors HSPGs and LRP1. Entry is independent of RAB5/RAB7 GTPases and clathrin in neurons. Reduced or mis-sorted cholesterol renders neurons vulnerable to tau entering the cytosol and permits seeded aggregation at concentrations of extracellular tau assemblies that fail to induce seeding in untreated cells. In contrast, exogenously supplied cholesterol acts as a barrier to entry and seeded aggregation. Our study enables investigation of tau entry distinct from uptake and should stimulate work to establish the relationship between dysregulated cholesterol and increased tau entry in human disease.

### Limitations of the study

The luciferase activity observed in our systems could result from a defect in clearance pathways rather than an increase in entry. This is mitigated by measurement of signal at early (1 h) time points. Tau may be taken up into astrocytes and microglia, but entry into these cell types was not addressed in this study. We used heparin-assembled filaments composed of a single tau isoform produced in *E. coli*. Recent cryoelectron microscopy structures reveal that heparin-assembled filaments are polymorphic and divergent to brain-origin assemblies (Zhang et al., 2019). Tau produced in *E. coli* does not retain native post-translational modifications. Future work is needed to determine whether there is similar behavior by authentically misfolded and post-translationally modified tau species. Finally, we use chemical and genetic treatments that may cause non-physiological changes in membrane cholesterol. Future work should determine whether the ranges of cholesterol concentrations observed in different disease or disease-risk states influence tau entry and seeded aggregation.

## STAR★Methods

### Key resources table

**Table:** 

REAGENT or RESOURCE	SOURCE	IDENTIFIER
**Antibodies**		

Rabbit anti-RAB5A	Proteintech	Cat# 11947-1-AP; RRID:AB_2269388
Rabbit anti-RAB7A	Proteintech	Cat# 55469-1-AP; RRID:AB_11182831
Rabbit anti-GFP	Proteintech	Cat# 50430-2-AP; RRID:AB_11042881
Rabbit anti-tubulin	Proteintech	Cat# 11224-1-AP; RRID:AB_2210206
Rabbit anti-LRP1	Abcam	Cat# ab92544; RRID:AB_2234877
Rabbit anti-VPS13D	Abcam	Cat# ab202285
Rabbit anti-NPC1	Abcam	Cat# ab134113; RRID:AB_2734695
Rabbit anti-histone H3	Abcam	Cat# ab176842; RRID:AB_2493104
Rabbit anti-EEA1	Abcam	Cat# ab2900, RRID:AB_2262056
Rabbit anti-NeuN	Abcam	Cat# ab177487, RRID:AB_2532109
Rabbit anti-pan-Tau	Agilent	Cat# A0024; RRID:AB_10013724
Mouse anti-VPS35	Santa Cruz Biotech	Cat# sc-374372; RRID:AB_10988942
Mouse anti-cyclophilin B	Santa Cruz Biotech	Cat# sc-130626; RRID:AB_2169421
Mouse anti-phospho-tau (AT8)	Thermo Fisher	Cat# MN1020; RRID:AB_223647
Mouse anti-GAPDH	Thermo Fisher	Cat# MA5-15738; RRID:AB_10977387
Mouse anti-LgBiT	Promega	Cat# N710A
Mouse anti-HiBiT	Promega	N/A
Chicken anti-MAP2	Abcam	Cat# ab5392; RRID:AB_2138153
Goat anti-mouse-HRP	Proteintech	Cat# SA00001-1; RRID:AB_2722565
Goat anti-rabbit-HRP	Proteintech	Cat# SA00001-2; RRID:AB_2722564

**Bacterial and virus strains**		

AAV1/2-hSyn-NPC1	This paper	N/A
AAV1/2-hSyn-eGFP-P2A-LgBiT-NLS	This paper	N/A
LV-PGK-NLS-eGFP-LgBiT	This paper	N/A
LV-EF-1α-mCherry-LgBiT	This paper	N/A
BL21(DE3) *E.coli*	Thermo Fisher	Cat# EC0114
DH10B *E.coli*	Thermo Fisher	Cat# EC0113

**Chemicals, peptides, and recombinant proteins**		

PitStop 2	Sigma-Aldrich	Cat# SML1169; CAS: 1332879-52-3
Heparin sodium salt	Sigma-Aldrich	Cat# H3393; CAS: 9041-08-1
25-hydroxycholesterol	Sigma-Aldrich	Cat# H1015; CAS: 2140-46-7
24(s)-hydroxycholesterol	Sigma-Aldrich	Cat# SML1648; CAS: 474-73-7
Methyl-β-cyclodextrin	Sigma-Aldrich	Cat# C4555; CAS: 128446-36-6
γ-cyclodextrin	Sigma-Aldrich	Cat# C4892; CAS: 17465-86-0
Efavirenz	Sigma-Aldrich	Cat# SML0536; CAS: 154598-52-4
Dimethyl amiloride	Sigma-Aldrich	Cat# A125; CAS: 1214-79-5
Surfen hydrate	Sigma-Aldrich	Cat# S6951; CAS: 329824572
Bafilomycin-A	Sigma-Aldrich	Cat# SML1661; CAS: 88899-55-2
Triton X-100	Sigma-Aldrich	Cat# X100
Filipin-III ready-made solution	Sigma-Aldrich	Cat# SAE0087
Lipofectamine 2000	Thermo Fisher	Cat# 11668019
Lipofectamine 3000	Thermo Fisher	Cat# L3000001
Lipofectamine RNAiMAX	Thermo Fisher	Cat# 13778075
Dyngo 4a	Abcam	Cat# Ab120689; CAS: 1256493-34-1
TopFluor cholesterol	Avanti Lipids	Cat# 810255P; CAS: 878557-19-8
OptiPrep Density Gradient Medium	Sigma-Aldrich	D1556
Polyethylene glycol 8000	Merck	Cat# PHR2894; CAS: 25322-68-3
0N4R-tau-GFP	Katsinelos et al., 2018	N/A
0N4R-P301S-tau-HiBiT	This paper	N/A
0N4R-P301S-tau	This paper	N/A
LgBiT protein	Promega	Cat# N2013
HiBiT peptide	Promega	N/A
GFP-HiBiT	This paper	N/A
Human Transferrin Alexa-647-conjugated	Thermo Fisher	Cat# T23366
Dimethyl sulfoxide (DMSO)	Sigma-Aldrich	Cat# D8418
Cultrex Poly-L-lysine	RnD Systems	Cat# 3438-100-01
Benzonase	Sigma-Aldrich	Cat# E1014-25KU

**Critical commercial assays**		

Nano-Glo Live Cell Assay System	Promega	Cat# N2013
PrestoBlue Viability Reagent	Thermo Fisher	Cat# A13261
TaqMan Universal PCR Master Mix	Applied Biosystems	Cat# 4305719
Q5 High-Fidelity PCR Kit	New England Biolabs	Cat# E0555L
Quick Ligation Kit	New England Biolabs	Cat# M2200L
PureLink HiPure Plasmid Maxiprep Kit	Thermo Fisher	Cat# K210016

**Experimental models: Cell lines**		

HEK 293T-NLS-eGFP-LgBiT	Generated in house	N/A
HEK 293T-mCherry-LgBiT-low	Generated in house	N/A
HEK 293T-mCherry-LgBiT-high	Generated in house	N/A
HEK 293T-REx-P301S-tau-venus	McEwan et al., 2017	N/A
HEK 293T-REx	Thermo Fisher	R71007
HEK 293T	ATCC	CRL-3216

**Experimental models: Organisms/strains**		

C57BL/6 mice	MRC-LMB	N/A
Thy1-hTau.P301S mice (CBA.C57BL/6)	MRC-LMB	Allen et al., 2002

**Oligonucleotides**		

See Figure S5 for qPCR primers	Sigma-Aldrich	N/A
OnTARGETplus non-targeting pool siRNA	Horizon Discovery	Cat# D-001810-10-05
OnTARGETplus *RAB5A* siRNA pool	Horizon Discovery	Cat# L-004009-00-0005
OnTARGETplus *RAB7A* siRNA pool	Horizon Discovery	Cat# L-010388-00-0005
OnTARGETplus *VPS13D* siRNA pool	Horizon Discovery	Cat# L-021567-02-0005
OnTARGETplus *VPS35* siRNA pool	Horizon Discovery	Cat# L-010894-00-0005
Accell *Rab5a* siRNA pool	Horizon Discovery	Cat# E-040855-00-0005
Accell *Rab7a* siRNA pool	Horizon Discovery	Cat# E-040859-01-0005
Accell *Lrp1* siRNA pool	Horizon Discovery	Cat# E-040764-00-0005
Accell *Npc1* siRNA pool	Horizon Discovery	Cat# E-047897-00-0005
Accell *Cypb* siRNA pool	Horizon Discovery	Cat# D-001920-20-05
eGFP FAM/ZEN probe FAM/CCGACA AGC/ZEN/AGAAGAACGGCATCAA	IDT	N/A
eGFP reverse qPCR primer ATGT TGTGGCGGATCTTGAAG	Sigma-Aldrich	N/A
eGFP forward qPCR primer CAA CAGCCACAACGTCTATATCAT	Sigma-Aldrich	N/A
PGK Forward WM17-1 TATCGAATT CTTCTACCGGGTAGGGGAGGCG	Sigma-Aldrich	N/A
PGK Rev WM17-2 GACTGGATCCA GGTCGAAAGGCCCGGAGATGA	Sigma-Aldrich	N/A

**Recombinant DNA**		

pAAV-hSyn-eGFP	Addgene	Cat# 50465
pAdDetlaF6 Helper	Addgene	Cat# 112867
pAAV 2/1	Addgene	Cat# 112862
pAAV 2/2	Addgene	Cat# 104963
pDONR221-NPC1-STOP	Addgene	Cat# 161461
pCRV-Gag-Pol	Prof. Stuart Neil, Kings College London	N/A
pMD2.G	Prof. Didier Trono, EPFL	Cat# 12259
pSMPP	Addgene	Cat# 104970
pRK172	Dr. Michel Goedert, MRC-LMB	N/A
NanoLuc-FRB-LgBiT	Promega	Cat# N2014
pNL1.1	Promega	Cat# N1001
pPMPP – created by replacing pSMPP promoter with PGK	This paper	N/A
pAAV-eGFP-P2A-LgBiT-nls	This paper	N/A
pAAV-NPC1	This paper	N/A
pMPP-NLS-eGFP-LgBiT	This paper	N/A
pSMPP-mCherry-LgBiT	This paper	N/A
pRK172-tau-HiBiT	This paper	N/A
pRK172-GFP-HiBiT	This paper	N/A

**Software and algorithms**		

Fiji	Fjii	https://fiji.sc
Prism 9	GraphPad	https://www.graphpad.com
BioRender	BioRender	https://biorender.com

### Resource availability

#### Lead contact

Further information and requests for resources and reagents should be directed to and will be fulfilled by the lead contact, William A. McEwan (wm305@cam.ac.uk).

#### Materials availability

All unique/stable reagents generated in this study are available from the lead contact with a completed materials transfer agreement.

### Experimental model and subject details

#### Mice

All animal work was licensed under the UK Animals (Scientific Procedures) Act 1986 and approved by the Medical Research Council Animal Welfare and Ethical Review Body. Wild-type C57BL/6 or Thy1-hTau.P301S (CBA.C57BL/6) mice were used in the study. Both males and females were used. Post-natal day 0 or day 1 pups were used for primary culture, and post-natal day 7 pups were used for slice culture. All animals were housed in pathogen-free conditions with daily routine and veterinary care procedures carried out.

#### Human iPSC-derived cortical neurons

Pluripotent iPSC cells were maintained in a tissue culture incubator at 37°C with 5% CO_2._ Cells were cultured on Vitronectin (Thermo Fisher, A14700) coated plates in feeder free conditions in complete TeSR-E8 Basal Medium (STEMCELL, 05991) and passaged using 0.5 mM EDTA. Differentiation of iNeurons was performed as previously described (Pawlowski et al., 2017) and can be found in method details. Differentiated iPSC-derived neurons were cultured in maintenance medium composed of Neurobasal medium (Gibco, 21103049), 1 mM Glutamax, 1X B27 (Gibco, 12587010), 10 ng/ml BDNF (Peprotech, 450-02), 10 ng/ml NT3 (Peprotech, 450-03), 1X antibiotic-antimycotic (Gibco, 15240096) and 1μg/ml doxycyline hyclate (Sigma-Aldrich, D9891-5G).

#### Cell lines

Human embryonic kidney cells were maintained in a tissue culture incubator at 37°C with 5% CO_2_. Cells were cultured in high-glucose DMEM (Gibco, 41966029) supplemented with 10% fetal bovine serum (Sigma-Aldrich, F4135) and 1% penicillin-streptomycin. Cells were maintained without antibiotic during AAV production and cell line generation.

### Method details

#### Primary neuron culture

Brains were removed from mice and primary neurons were isolated from the cortices and hippocampi as previously described (Beaudoin et al., 2012). The protocol was adapted to produce pooled hippocampal and cortical cultures. Cortices and hippocampi from 6 mice were isolated in Hibernate-A medium (Gibco, A1247501) and pooled in a 15 ml conical tube. Tissue was then washed gently twice with 10 ml Hibernate-A. Media was removed and replaced with 4.5 ml Hibernate-A plus 500 μl 10X Trypsin protease solution (Gibco, 15090046) and incubated at 37°C for 20 min. During incubation, a 9-inch glass cotton plugged Pasteur pipette (Thermo Fisher Scientific, 13-678-8B) was flame polished until the tip resembled the diameter of a P1000 pipette tip. The trypsinized tissue was washed twice with room temperature Hibernate-A followed by a single wash with pre-warmed plating medium composed of Neurobasal Plus (Gibco, A3582901) 1mM GlutaMAX (Gibco, 35050061), 10% horse serum, 1% penicillin-streptomycin and 1x B-27 Plus (Gibco, A3582801). The tissue was triturated exactly 9 times before straining through a 70 μm cell strainer. Cells were then counted with trypan blue staining. 30,000 cells were seeded per well into a white (Greiner bio-one) poly-L-lysine coated (RnD Systems, 3438-100-01) 96-well plate in plating medium for 4 h before a complete media change to maintenance medium (plating medium devoid of serum) and maintained in a tissue culture incubator with at 37°C with 5% CO_2_. Media was topped up with 50% of volume on DIV 2.

#### Organotypic hippocampal slice culture

Slices were prepared from P301S tau transgenic pups aged 7 days as we previously described (Miller et al., 2021). Brains were extracted and maintained in slicing medium (EBSS +25 mM HEPES) on ice. Brains were bisected along the midline using a sterile scalpel and the medial surface attached to the stage of a Leica VT1200S Vibratome using cyanoacrylate (Loctite). Sagittal slices of 300 μm thickness were removed and the hippocampus was dissected using sterile needles. Slices were maintained on membranes with 0.4 μm pore size (Millipore) in 6 well plates with 1 ml Slice Culture Medium as follows: 50% MEM with GlutaMAX (Gibco, 41090036), 18% EBSS (Gibco, 24010043), 6% EBSS + D-glucose, 1% penicillin-streptomycin (Gibco, 15140122), 0.06% nystatin (Gibco, 15340029), and 25% horse serum (Gibco, 26050070). Slices were maintained at 37°C and 5% CO_2_ in a humid atmosphere.

#### Differentiation of iPSC-derived cortical neurons

After 1–2 weeks of culture of pluripotent iPSCs (see experimental models and subject details), cells were dissociated into single cells with accutase (Gibco, A1110501) and plated on 0.1 mg/mL Poly-D-Lysine (Gibco, A3890401) + Geltrex (Gibco, A1413302) coated plates at a density of 25,000 cells per cm^2^. Differentiation was induced 24 h after plating (Day 0) in differentiation medium composed of DMEM/F12 (Gibco, 21331-020), 1 mM Glutamax, 1X non-essential amino acids (Gibco, 1140035), 1X N2 (Gibco, 17502001), 50 μM 2-mercaptoethanol, 1X antibiotic-antimycotic and 1 μg/ml doxycyline hyclate. From Day 2 the media was switched to maintenance medium. On Day 4 the neurons were replated into 24 and 96 well-plates at a density of 100,000 cells per well or 50–80,000 cells per well, respectively. Media was replaced with fresh maintenance medium each day until Day 7 and then every 48 h onwards.

#### Protein production

Human 6xHis-P301S-0N4R-tau-HiBiT or 6x-His-GFP-HiBiT was cloned into the bacterial expression vector pRK172, expressed in BL21 DE3 competent *E. coli*, and purified as previously described in 4 X 1L format (Katsinelos et al., 2018). Protein expression was induced with 500 μM IPTG overnight (12–16 h) at 16°C. Cells were pelleted (17000 x g, 3 min) and lysed in lysis buffer (1 mM benzamidine, 1 mM PMSF, 1X protease inhibitors (Thermo Fisher, 78440), 14 mM β-mercaptoethanol, 300 mM NaCl, 25 mM HEPES, 30 mM imidazole, 1% NP-40). Lysates were cleared via ultracentrifugation (100,000 x g, 50 minutes, 4°C) and his-tagged protein purified on the AKTÄ Pure via the HisTrap HP column according to manufacturer instructions (GE Healthcare). Fractions were analyzed by SDS-PAGE. GFP-HiBiT fractions were pooled in PBS and concentrated, followed by snap freezing and long-term storage at −80°C. Tau fractions were second round purified via size exclusion chromatography on the Superdex 200 column according to manufacturer instructions (GE Healthcare). Fractions were then pooled in PBS with 1 mM DTT, concentrated to >3 mg/ml and snap frozen in liquid nitrogen followed by long-term storage at −80°C. 0N4R-tau-GFP protein was expressed in SF9 cells as previously described (Katsinelos et al., 2018).

#### Tau aggregation

60μM tau monomer was incubated with 20 μM heparin, 2 mM DTT and 1X protease inhibitors in PBS for 24–72 h at 37°C shaking at 250 RPM. Tau aggregation was quantified by Thioflavin T (ThT, Thermo Fisher, T3516) fluorescence readout (excitation 440 nm; emission 510 nm) in a ClarioSTAR plate reader (BMG Labtech) with 5 μM tau aggregates and 15 μM ThT.

#### AAV production and titer

eGFP-P2A-LgBiT-NLS or Human NPC1 was cloned into the pAAV-hSyn-eGFP vector genome plasmid via PCR to generate pAAV-hSyn-GPLN or pAAV-hSyn-NPC1 by replacing eGFP. Chimeric particles of adeno-associated virus produced with capsids of types 1 and 2 (AAV1/2) were prepared as previously described (Cheng et al., 2018). 7 μg pAAV-hSyn-eGFP or pAAV-hSyn-NPC1, 3.5 μg pAAV2/1, 3.5 μg pAAV2/2 and 20 μg pAdDeltaF6 helper plasmid per plate were co-transfected in 10 × 15 cm cell culture dishes of 60% confluent HEK293 cells via polyethylenimine (PEI). After 48–60 h, the medium was pooled into a separate container, cells were harvested, pelleted (3000 x g, 10 min), and lysed in 10 mL total AAV lysis buffer (20 mM Tris pH 8.0, 1 mM MgCl_2_, 150 mM NaCl). The resuspended pellet was frozen at −80°C for future use. NaCl (0.93 g) and 10 mL of 40% polyethylene glycol 8000 (PEG) (Merck) was added per 40 mL media and incubated on ice overnight. Media: PEG solutions were then centrifuged (6600 RPM, 4°C). Pellets were resuspended in 4 mL total AAV lysis buffer and pooled with the cell pellet. The solution was thawed and incubated with 1 mM MgCl_2_ and 100 U benzonase (Sigma-Aldrich) for 15 min at 37°C. The solution was then freeze thawed 3 times before a final benzonase digestion with an additional 100 U. The solution was then centrifuged (3000 x g, 20 min, 4°C) and viral supernatant was next subjected to iodixanol (Optiprep) (Sigma-Aldrich) gradient ultracentrifugation. Iodixanol was layered into a Beckmann ultracentrifuge tube (Beckmann, 362183) with the following solutions: 6 mL of 17% (5mL 10X PBS, 0.05mL 1 M MgCl_2_, 0.125mL 1 M KCl, 10mL 5 M NaCl, 12.5mL Optiprep, H_2_0 to 50mL) 6 mL of 25% (5mL 10x PBS, 0.05mL 1 M MgCl_2_, 0.125mL 1 M KCl, 20mL Optiprep, 0.1mL of 0.5% phenol red, H_2_0 to 50mL), 5 mL of 40% (5mL 10x PBS, 0.05mL 1 M MgCl_2_, 0.125mL 1 M KCl, 33.3mL Optiprep and H_2_0 to 50mL) and 6 mL of 60% (0.05mL 1 M MgCl_2_, 0.125mL 1 M KCI, 50mL Optiprep, 0.025mL 0.5% phenol red). Virus solution was then layered on top of the 17% layer and ultracentrifuged (68,000 RPM, 17°C, 1 h 10 min). The 40% iodixanol fraction was isolated and concentrated to ∼100 μL in PBS. Virus was frozen at −80°C in single use aliquots. Genome titers were determined via qPCR (sequences available in the key resources table) or via SDS-PAGE densitometry with known titer AAV as a reference. qPCR was performed using TaqMan Universal PCR Master Mix (Applied Biosystems) according to manufacturer instructions, and purity determined via SDS-PAGE followed by Coomassie staining.

#### SDS-PAGE and western blotting

Samples for Western blot were lysed in appropriate volumes of 1X RIPA buffer (Sigma, R0278) with 1X protease inhibitors, lysates cleared via centrifugation (max RPM, 10 min at 4°C) and resuspended with appropriate volume of 4X NuPAGE LDS sample buffer (Thermo Fisher, NP0007) with 2 mM β-mercaptoethanol and boiled at 100°C for 10 min. Samples were subjected to SDS-PAGE using NuPAGE Bis-Tris 4-12% gels (Thermo Fisher, NP0324BOX) and electroblotted onto a 0.2 μm PVDF membrane using the Bio-Rad Transblot Turbo Transfer System. Transferred membranes were blocked in blocking buffer (5% milk in 0.1% tween 20 in 1X TBS (TBS-T)) for 1 h at room temperature, and incubated with primary antibody at desired concentration diluted in blocking buffer at 4°C overnight. Membranes were repeatedly washed with TBS-T (3 × 5 min) and incubated with secondary HRP- or alexa-flour-conjugated antibody (Thermo Fisher) for 1 h at room temperature. Membranes were washed (4 × 5 min) with TBS-T before being incubated with HRP substrate (Millipore, WBKLS0500) and membranes imaged using a ChemiDoc gel imager (Bio-Rad). Subcellular fractionation of the nucleus and cytosol prior to western blotting was performed via the REAP method, as previously described (Suzuki et al., 2010).

#### iNeuron qPCR

Cells were collected on Day 0, Day 4, Day 7 and Day 14 for RNA extraction using Qiagen RNAeasy plus kit (74134). 350ng of RNA from each sample was converted into cDNA using Quantitect reverse transcription kit (Qiagen, 205313). Sample was diluted to 5 ng/μL and used in a 5 μL reaction with Luna universal qPCR master mix (New England biolabs, M3003L) and primers designed to detect a range of genes (Figure S5). qPCR was performed on the QuantStudio5 (Thermo Scientific). All samples were analyzed in technical triplication from 3 independent differentiations. qPCR data was normalized to housekeeping gene 18S and results analyzed using the ΔΔCt method.

#### Lentiviral transduction

Lentivirus was produced in HEK293 cells via co-transfection of pcRV-Gagpol, pMD2.G and the pPMPP genome vector via lipofectamine 3000 (Thermo Fisher) according to manufacturer instructions. Supernatant containing packaged lentivirus was harvested after ∼24 h and ∼48 h and pooled together. Lentivirus containing supernatant was centrifuged at 500 x g for 5 min to pellet suspended cells and debris. The supernatant was transferred to a new tube and either transduced immediately, or aliquoted and frozen at −80°C for future use. Target cells were infected with a titration of lentivirus-containing supernatant supplemented with 5 μg/mL polybrene (Sigma-Aldrich, TR-1003) in complete DMEM without penicillin-streptomycin. 48-h post infection, cells were selected with puromycin dihydrochloride in complete DMEM at a final concentration of 1–10 μg/mL (Gibco, A1113803). Media was changed every 48–72 h and fresh puromycin supplied. Individual colonies of resistant cells were expanded and expression of the gene of interest was confirmed via Western blot and/or fluorescence microscopy.

#### Genetic knockdown

All siRNA used in the study are depicted in the key resources table. Human onTARGETplus siRNA were diluted to 20 μM stock in RNase-free water and cells were transfected with 10 nM siRNA for 3 days with Lipofectamine RNAiMAX transfection reagent (Thermo Fisher) in 6 well format. Knockdown cells were re-plated at day 3, and tau entry assayed at day 4. Mouse Accell SMARTpool siRNA were diluted to 100 μM in RNase-free water and added directly to neurons on DIV 4 at a final concentration of 1 μM. Tau entry assays were performed 72 h post-knockdown.

#### HEK293T tau entry assay

LgBiT expressing HEK293T cells were seeded 2 × 10^4^ cells per well into white 96-well plates (Greiner bio-one, 655098) coated with poly-L-lysine (Sigma, P4707) in complete DMEM. 12–16 h later, the medium was replaced with 50 μL serum free CO_2_ independent medium (Thermo Fisher, 18045088) supplemented with 1 mM sodium pyruvate, 1% penicillin-streptomycin, 1 mM GlutaMAX and 50 μL tau-HiBiT solution added to final concentration in 100 μL. Cells were pre-treated for the indicated times with drugs, equivalent volume of solvent control, or supplemented with lipofectamine 2000 (Thermo Fisher). After tau incubation, media was aspirated and cells washed once with PBS. PBS was aspirated and replaced with CO_2_ independent medium plus live cell substrate according to manufacturer instructions (Promega, N2013). The cells were incubated for 5 min and immediately loaded onto the ClarioSTAR plate reader where luminescent signal was quantified at 37°C. Where relevant, 0.25% trypsin -EDTA solution (Gibco, 25200056) was added to a final concentration of 0.125% for 5–10 min at 37°C prior to signal acquisition. RLU data was normalized to viable cells per well acquired from a PrestoBlue viability assay when expressed as Cytosol entry (RLU/cell).

#### Neuronal tau entry assay

Primary neurons were infected at DIV 2 with AAV1/2 hSyn::-eGFP-P2A-LgBiT-nls particles at a multiplicity of 50,000 genome copies per cell to express LgBiT and/or hSyn::-NPC1 particles of a multiplicity of 100,000 genome copiers per cell to express NPC1 when required. On day of assay, DIV 7 neurons were 100% media changed to fresh maintenance medium with desired concentration of tau-HiBiT. Neurons were pre-treated by freshly changing to medium supplemented with desired concentration of compound or equivalent volume solvent for the required amount of time, followed by addition of tau-HiBiT to final desired concentration (without removing compound). Signal quantification and trypsin incubation was performed as described for the HEK293T tau entry assay. iPSC-derived cortical neurons were infected with AAV1/2 hSyn::-eGFP-P2A-LgBiT-nls particles at a cumulative multiplicity of 200,000 genome copies per cell on Day 5 (50,000), 6 (50,000) and 9 (100,000) of culture. Pre-treatments, entry assays and quantification were performed on day 14 as described for primary neurons.

#### PrestoBlue viability assay

After tau entry signal acquisition, PrestoBlue Cell Viability Reagent (Thermo Fisher) was added to cells according to manufacturer instructions and incubated for 42 min at 37°C and 5% CO_2_. The plate was loaded into the ClarioSTAR plate reader and fluorescence intensity quantified (excitation 540–570 nm; emission 580–610 nm). Total viable cells per well were calculated using a standard curve of known viable cells per well and adjusted for background fluorescence.

#### HEK293T seeding assay

The seeding assay was performed as previously described (McEwan et al., 2017). Briefly, 20,000 cells were seeded into poly-L-lysine (Sigma, P4707) coated black 96-well plates in 50 μL OptiMEM (Thermo Fisher, 31985062). Tau assemblies were diluted in OptiMEM and 50 μl added to cells to final concentration in the presence or absence of 1% Lipofectamine 2000 (Thermo Fisher). Cells were incubated at 37°C and 5% CO_2_ for 1 h before the addition of 100 μL complete medium to stop the transfection process. Cells were incubated at 37°C in an IncuCyte S3 Live-Cell Analysis System for 48–72 h after the addition of tau assemblies and aggregation quantified in Fiji.

#### Neuronal seeding assay

Primary neurons were supplemented with a final concentration of 100 nM tau assemblies at DIV 7 in maintenance medium and incubated at 37°C and 5% CO_2_ until DIV 14. Compounds or equivalent volume solvents were diluted in maintenance medium on DIV 6 or DIV 7 and pre-treated for depicted times where relevant. Compounds were retained on the cells throughout the duration of the assay. For NPC1 studies, neurons were transduced with 100,000 genome copies per cell of AAV1/2 hSyn::-NPC1 at DIV 3 prior to seeding on DIV 7. At DIV 14, cells were fixed with methanol, nuclei stained with DAPI and epitopes immunofluorescently labeled with MAP2 and AT8 antibodies. Stained neurons were subjected to high-content imaging and tau aggregation quantified. AT8-positive puncta per field were normalized to cell count per field.

#### OHSC seeding assay

Compounds or equivalent volume solvent was diluted in 1 mL culture medium on day 6 and applied to the underside of the slices for 1 day before addition of 100 nM tau on day 7. 3 days later, media was removed and fresh medium added without drug or tau. 3 weeks post-challenge, OHSCs were fixed in 4% paraformaldehyde, nuclei stained with DAPI, and MAP2, phopsho-tau (AT8) and tau (DAKO) epitopes immunofluorescently labeled. Stained slice images were acquired using an SP8 Lightning Confocal Microscope (Leica) with either a 20X or 63X objective lens.

#### Plasmid-based endosomal lysis assay

HEK293T cells were plated at 10,000 cells per well in 96 well plates. The next day, media was exchanged for serum-free complete medium with pNL1.1 plasmid at 10 ng/μL. Recombinant tau monomers or heparin-assembled tau were added to the media. Lipofectamine 2000 and endosome-destabilizing adenovirus type 5 (ViraQuest) were added as positive controls for entry. Plates were examined for NanoLuc luminescence after 24 h on the ClarioSTAR plate reader.

#### HEK293T transferrin uptake

500,000 HEK293T cells were suspended in serum-free complete medium and pre-treated with compounds for 30 min at 37°C and 5% CO_2_. 10 μg/mL Alexa-Flour-647-conjugated human transferrin (Thermo Fisher) was added to the cell suspension and incubated on ice for 5 min, followed by 10 min at 37°C and 5% CO_2_. Cells were pelleted (500 x g, 5 min), acid washed twice (100 mM glycine, 150 mM NaCl pH 2.5 in PBS), fixed in 4% PFA (10 min at room temperature) and subsequently resuspended in FACS buffer (1% bovine serum albumin in PBS). Transferrin uptake was then quantified via flow cytometry (CytoFLEX flow cytometer, Beckman Coulter). 10,000 events were recorded per condition. Cell sorting was performed on the BD FACSMelody Cell Sorter System (BD Biosciences).

#### Neuron transferrin uptake

DIV 7 primary neurons (800,000 cells/well) or day 14 iPSC-derived human neurons (760,000/well) in 6 well format were washed with PBS followed by pre-treatment for 1 h with compounds or equivalent volume solvent in maintenance medium. Cells were then incubated with 10 μg/mL AlexaFluor-647-conjugated human transferrin (Thermo Fisher) for 10 min at 37°C and 5% CO_2_. Media was aspirated and cells submerged in acid wash (100 mM glycine, 150 mM NaCl pH 2.5 in PBS) for 60 s. Cells were washed with PBS and detached with accutase for 15 min in a tissue culture incubator. Cells were pelleted (500 x g, 5 min) and fixed in 4% PFA for 10 min at room temperature. Fixed cells were pelleted (500 x g, 5 min) and resuspended in FACS buffer. Transferrin uptake was then analyzed via flow cytometry (CytoFLEX flow cytometer, Beckman Coulter). 10,000 events were recorded per condition.

#### Immunofluorescence

Media from cells was aspirated and cells were rinsed once with PBS. Sample was then fixed in 4% PFA in PBS (Thermo Scientific, J19943.K2) for 10 min at room temperature. PFA was aspirated and cells were rinsed 3 times with PBS followed by permeabilization with 0.1% Triton X-100 in PBS for 10 min at room temperature. Cells were then washed 3 times with PBS and blocked with 2% BSA in PBS (IF block) for 1 h at room temperature. Primary antibody was diluted to required concentration in IF block and incubated overnight at 4°C. Antibody was removed, cells were rinsed 3 times with PBS and incubated with Alexa-Fluor conjugated secondary antibody (Thermo Fisher) in IF block for 1 h at room temp. Secondary antibody was removed, cells rinsed with PBS 3 times and imaged via fluorescence microscopy. Filipin III staining was performed according to manufacturer instructions (Thermo Fisher). For methanol fixation of neurons, cells were fixed and permeabilized with ice-cold methanol on ice for 3 min. 3 half washes with PBS (1X volume added, 0.5X total volume removed) were then performed followed by aspiration and 3 full washes with PBS. Permeabilized cells were then blocked and stained as described.

#### Tau-GFP uptake assay

0N4R-Tau-GFP assemblies were incubated on HEK293T cells or neurons in complete medium or maintenance medium respectively, for 1 h. Cells were then washed twice with PBS, followed by fixation with 4% PFA (HEK293T) or methanol (neurons) and processed for downstream immunostaining as described.

#### Transmission electron microscopy

Formation of Tau-HiBiT fibrils was confirmed by uranyl acetate negative stain transmission electron microscopy at the Cambridge Advanced Imaging Centre as previously described (Goedert et al., 1992).

#### Image analysis

Confocal images were acquired on a SP8 Lightning Confocal Microscope (Leica). Other fluorescence images were acquired on a Ti2-E High Content Microscope (Nikon) or the IncuCyte S3 Live-Cell System. Puncta corresponding to seeded aggregation were quantified in Fiji using the ComDet plugin (Schindelin et al., 2012). Total puncta per field was normalized to cell count per field via DAPI staining. AT8 staining in OHSCs was segmented into a binary threshold and fields of 150 μm × 150 μm were analyzed for % AT8 positive area.

### Quantification and statistical analysis

All statistical analyses were performed via GraphPad Prism software. Differences between multiple means were tested by one-way ANOVA, followed by Tukey’s post hoc test unless otherwise indicated in the figure legend. Differences between two means were tested by unpaired Student's t test with Welch’s correction. All data represent mean values ± the standard error of the mean (SEM) with the following significances ^ns^ p > 0.05, ^∗^p < 0.05, ^∗∗^p < 0.01, ^∗∗∗^p < 0.001, ^∗∗∗∗^p < 0.0001.

## Data Availability

• All data reported in this paper will be shared by the lead contact upon request. • This paper reports no original code. • Any additional information required to reanalyze the data reported in this paper is available from the lead contact upon request.

## Supplemental information

Document S1. Figures S1–S7

Document S2. Article plus supplemental information
