# Supplementary material for: Cholesterol determines the cytosolic entry and seeded aggregation of tau
Source: Cell Rep. 2022 May 6;39(5):110776. doi: 10.1016/j.celrep.2022.110776 (PMC9108550; doi:10.1016/j.celrep.2022.110776)
Supplement: Document S1. Figures S1–S7 [file mmc1.pdf]

Cell Reports, Volume *39*

Supplemental information

Cholesterol determines the cytosolic entry and seeded aggregation of tau

Benjamin J. Tuck, Lauren V.C. Miller, Taxiarchis Katsinelos, Annabel E. Smith, Emma L. Wilson, Sophie Keeling, Shi Cheng, Marina J. Vaysburd, Claire Knox, Lucy Tredgett, Emmanouil Metzakopian, Leo C. James, and William A. McEwan

Figure S1 (related to Figure 1)

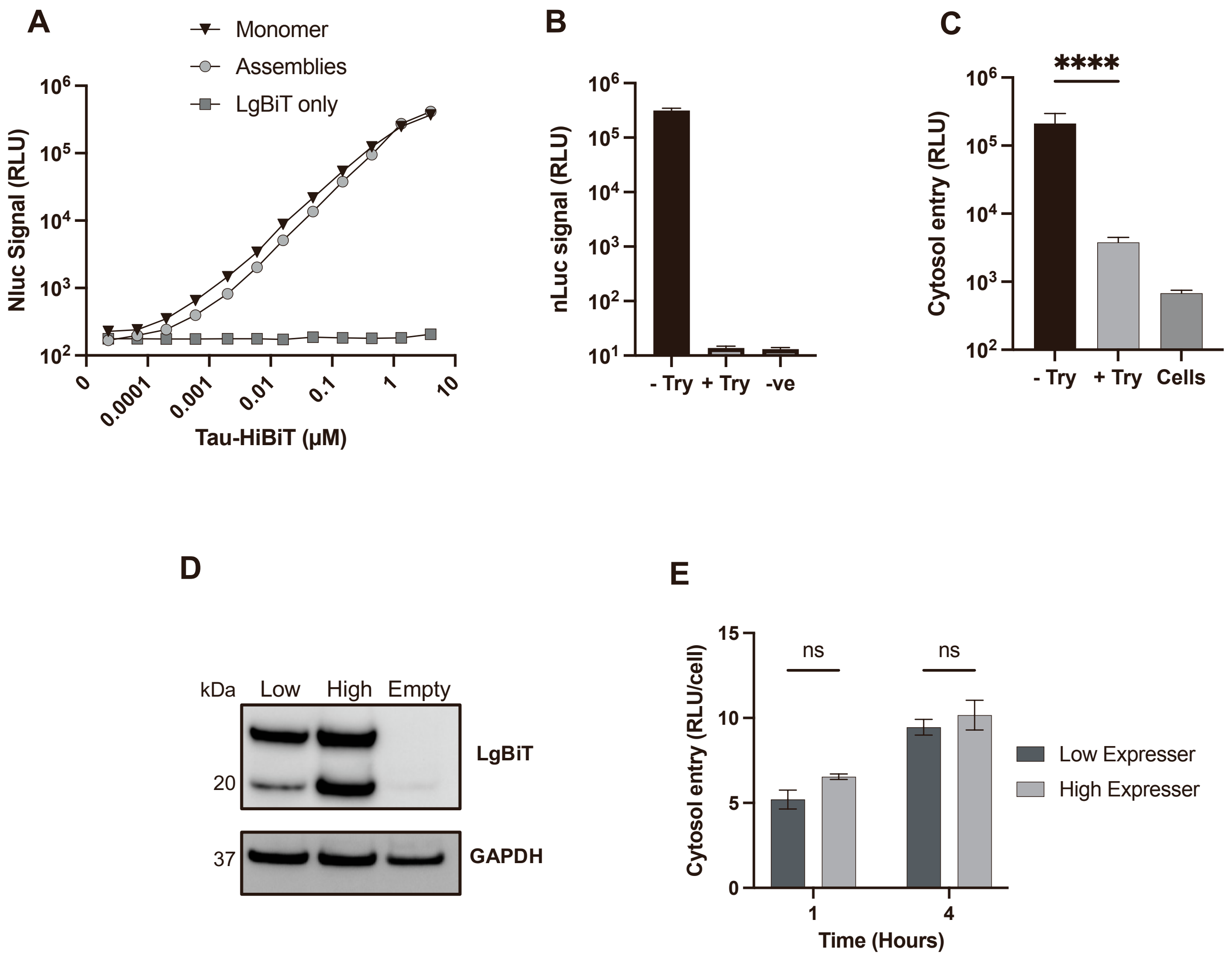

Figure S1 (related to Figure 1): Extracellular signal is sensitive to trypsin and LgBiT concentration is not limiting.
A) Titration of tau-HiBiT monomer or heparin-induced assemblies in the presence of recombinant LgBiT; n=3. Error denotes SEM.
B) In vitro (cell free) reconstitution of NanoLuc with 4 µM tau-HiBiT assemblies complexed with recombinant LgBiT in the presence or absence of trypsin (Try); n=2-9. Error denotes SEM.
C) 10 minutes trypsin protease treatment of HEK-LgBiT after incubation with 250 nM tau-HiBiT assemblies for 1 h, n=6. Error denotes SEM. **** $p < 0.0001$ by one-way ANOVA with Tukey's multiple comparisons.
D) Western blotting of mCherry-LgBiT HEK cells with low and high expression. The upper band corresponds to mCherry-LgBiT (43 kDa) and the lower band LgBiT (19 kDa). Empty refers to 293T cell lysate only.
E) Signal following application of 10 nM tau-HiBiT assemblies to low and high expressing mCherry-LgBiT cells over 4 h, n=4. Error denotes SEM.

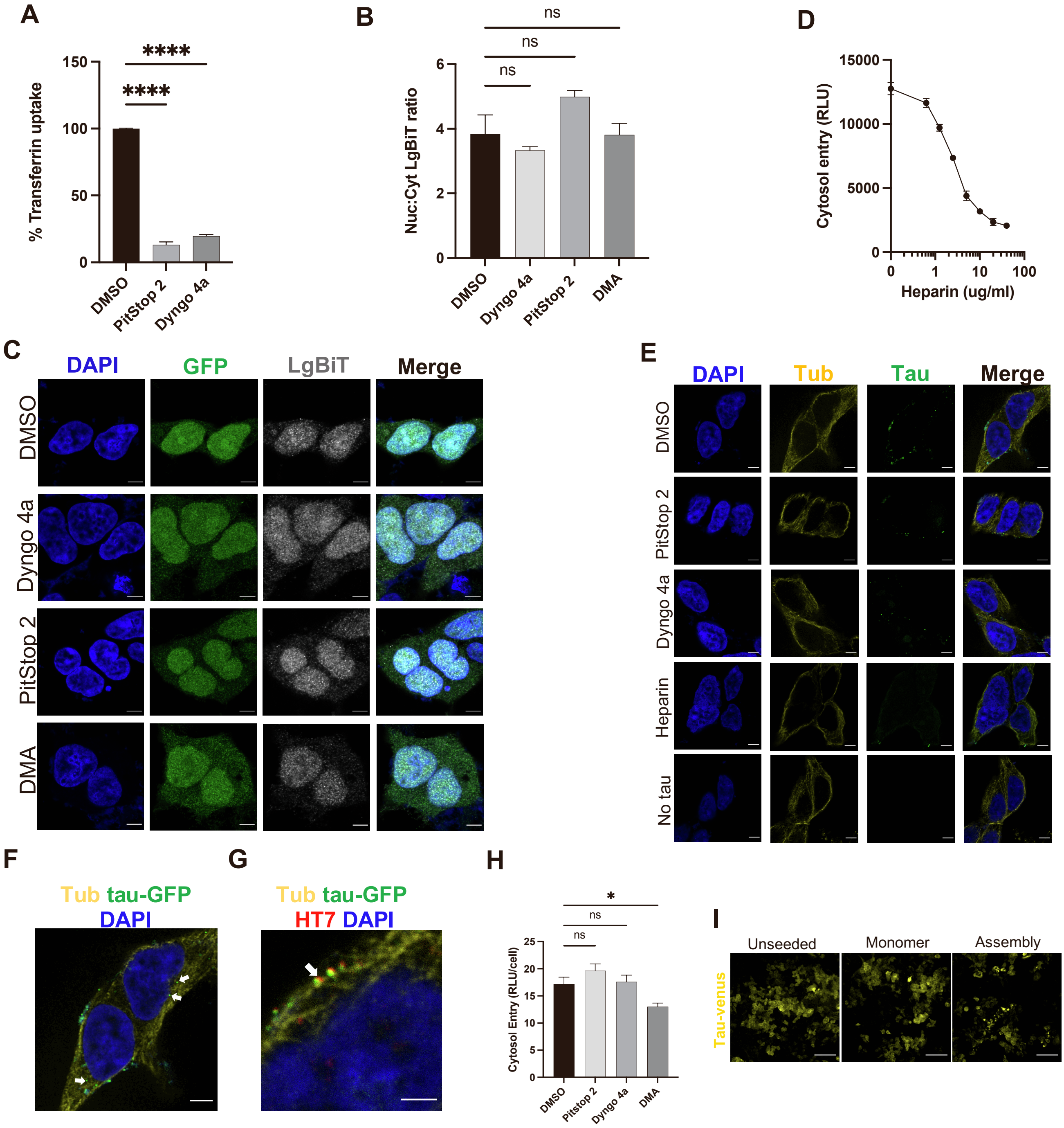

Figure S2 (related to Figure 2): Pathway inhibitors impair tau-GFP uptake and do not affect LgBiT localisation.
A) Uptake of 10 µg/ml alexa-fluor-647-conjugated human transferrin to HEK293T cells, analysed by flow cytometry. Cells were pre-treated for 30 minutes with PitStop 2 or Dyngo 4a (both 20 µM) prior to addition of transferrin for 15 minutes. 1x10^4 events were recorded from 3 independent experiments. Error denotes SEM. **** $p < 0.0001$ by one-way ANOVA with Tukey's multiple comparisons.
B) Quantification of nuclear:cytosol ratio of LgBiT from confocal microscope images shown in (**C**) after treatment with Dyngo 4a (20 µM), PitStop 2 (20 µM), DMA (500 µM) or solvent (DMSO) for 1 h. Error denotes SEM.
C) Confocal microscopy of HEK-NGL cells treated with drug or solvent as described in (**B**) and immunostained for GFP and LgBiT. Scale bars, 5 µm.
D) 1 h tau entry assay with 50 nM tau-HiBiT assemblies in HEK-NGL cells following pre-treatment with increasing concentrations of heparin for 1 h; n=3. Error denotes SEM.
E) Confocal microscopy of HEK 293T cells pre-treated with PitStop 2 (20 µM), Dyngo 4a (20µM), Heparin (40 µg/ml) or solvent (DMSO) for 1 h followed by incubation with 50 nM tau-GFP assemblies for 1 h. Scale bars, 5 µm.
F) Zoomed in view of cells from panel (**E**) demonstrating intracellular tau-GFP assemblies (white arrows).Scale bar, 5 µm.
G) Tau-GFP assemblies co-localised with human tau HT7 antibody. Scale bar, 2 µm.
H) Entry of 50 nM tau-HiBiT monomer to HEK-NGL cells after pre-treatment with PitStop 2 (20 µM), Dyngo 4a (20 µM), DMA (200 µM) or solvent control (DMSO) for 1 h; n=6. Error denotes SEM. * $p < 0.05$ by unpaired t test.
I) Fluorescence microscope images of P301S-tau-venus biosensor cell line 72 h after challenge with 2 nM of tau monomer or assemblies in the presence of 1% lipofectamine. Scale bars, 50 µm.

Figure S3 (related to Figure 3)

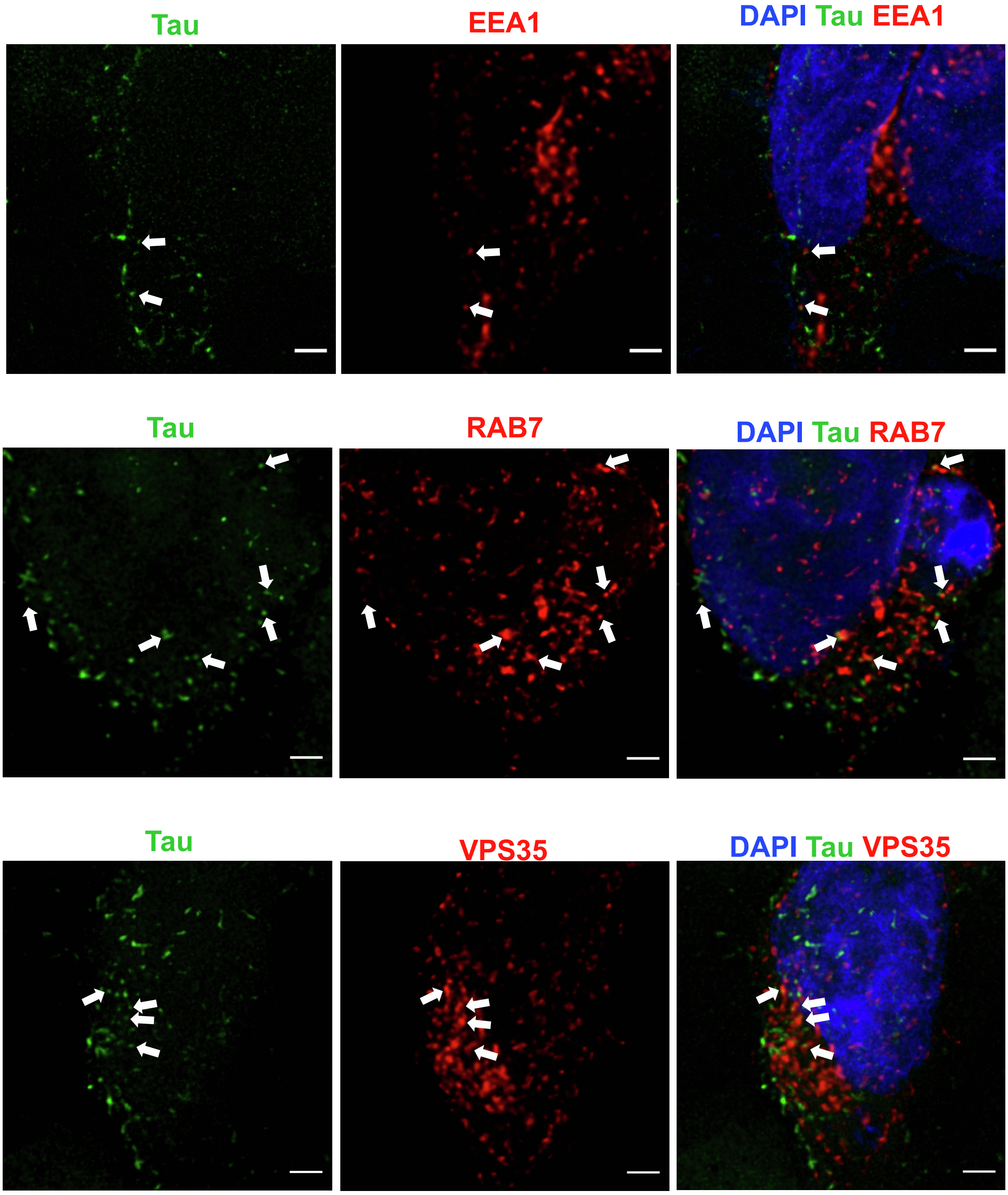

Figure S3 (related to Figure 3): Tau-GFP assemblies co-localise with endolysosomal markers. Confocal microscopy (z-stack) of HEK293T cells after a 1 h uptake assay with 200 nM tau-GFP assemblies. Cells were fixed and stained for early endosomal marker EEA1, late endosome marker RAB7 or retromer associated VPS35. White arrows indicate areas with colocalization. Scale bars, 5 µm.

Figure S4 (related to Figure 4)

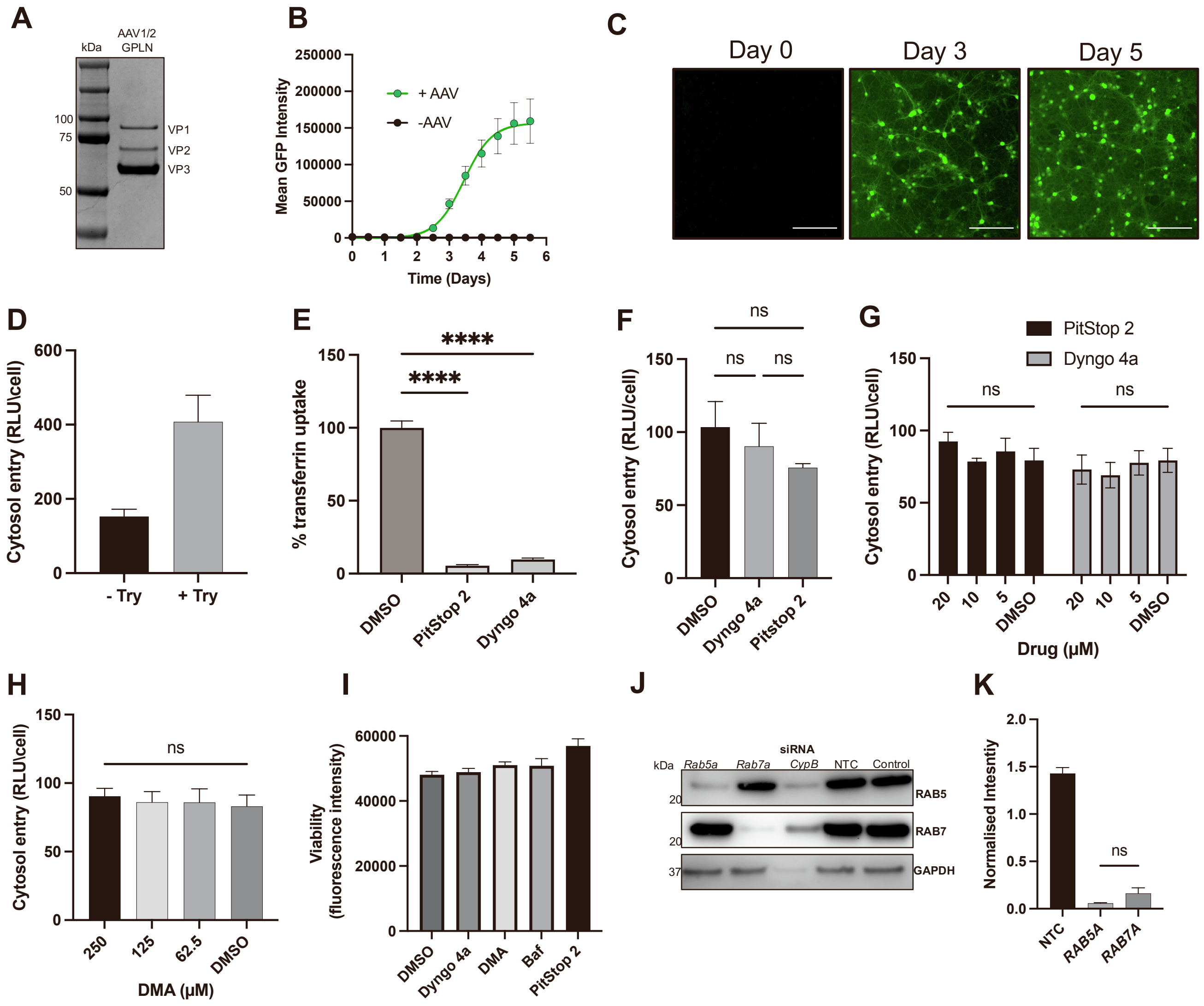

Figure S4 (related to Figure 4): Neuronal entry signal is not reduced by trypsin and monomeric entry is unaffected by pathway inhibitors.

A) Coomassie staining of purified AAV1/2-hSyn-GPLN after SDS-PAGE. Viral proteins VP1, VP2, and VP3 are labelled.

B) Time-course of GFP expression of primary mouse neurons following transduction with AAV1/2-hSyn-GPLN at a multiplicity of 50,000 genome copies/cell over the course of 5 days; n=6. Error denotes SEM.

C) Representative images of AAV transduced neurons from panel **B** at depicted time points. Scale bars, 200 μm.

D) Treatment of GPLN-neurons with or without trypsin (Try) for ten minutes prior to signal acquisition after incubation with 50 nM tau-HiBiT assemblies for 1 h. In the +Try condition we observed rounding of cells, likely amplifying luminescent signal; n=3, N=2 independent experiments. Error denotes SEM.

E) Transferrin uptake assay in DIV 7 primary neurons with 10 μg/ml Alexa-Fluor-647-conjugated human transferrin. Cells were pre-treated for 1 h with PitStop 2 or Dyngo 4a (both 20 μM) prior to addition of transferrin for 15 minutes. 1×10^4 events were recorded from 3 independent experiments. Error denotes SEM. **** $p < 0.0001$ by one-way ANOVA with Tukey's multiple comparisons.

F) Tau entry in DIV 14 GPLN-neurons after pre-treatment with Dyngo 4a (20 μM), PitStop 2 (20 μM) or solvent DMSO for 1 h followed by a 1 h entry assay with 50 nM tau-HiBiT assemblies. Neurons were transduced at DIV 9; n=3. Error denotes SEM.

G and H) Entry of 50 nM tau-HiBiT monomers 1 h after challenge with indicated concentrations of PitStop 2, Dyngo 4a (**G**) or DMA (**H**). GPLN-neurons were pre-treated for 1 h prior to assay; n=3, N=3 independent experiments. Error denotes SEM.

I) Presto blue viability assay of GPLN-neurons after tau entry assay and Dyngo 4a (40 μM), DMA (250 μM), Bafilomycin-A (200 nM), PitStop 2 (40 μM), or solvent control (DMSO) treatment throughout, showing no reduction in viability with any drug compared to control; n=3. Error denotes SEM.

J and K) Full uncropped western blot from **Fig. 4** (main text) (**J**), and quantification of band densitometry (**K**) from 3 independent experiments. Error denotes SEM.

Figure S5 (related to Figure 4)

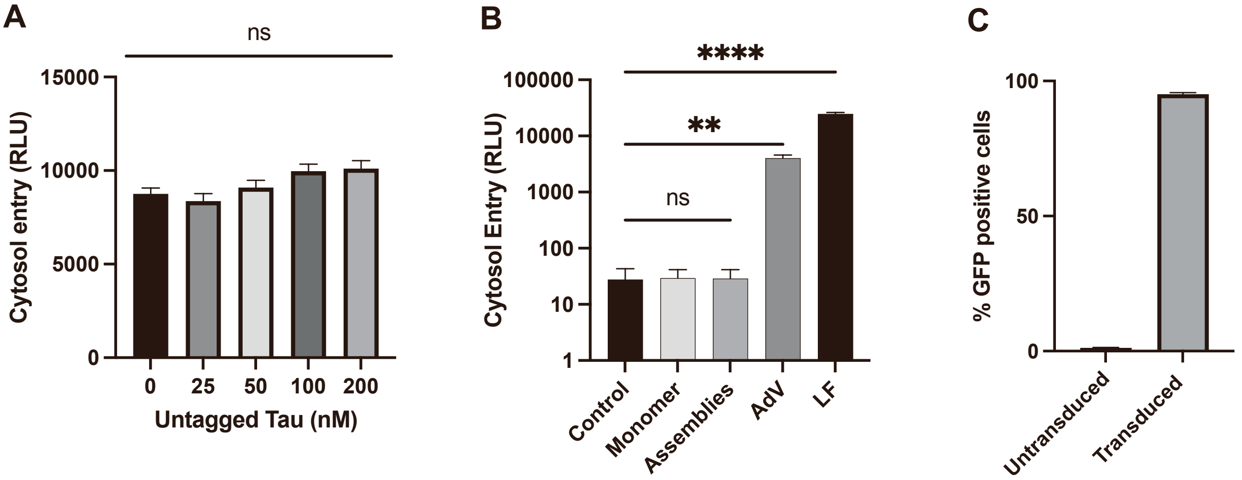

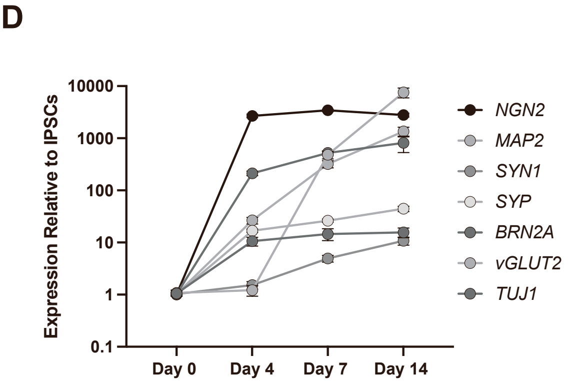

E

Name	Sequence (5'-3')
BRN2_fw	ACCCGCTTTATCGAAGGCAA
BRN2_rv	CCTCCATAACCTCCCCCAGA
VGLUT2_fw	GTAGACTGGCAACCACCTCC
VGLUT2_rv	CCATTCCAAAGCTTCCGTAGAC
TUBB3_fw	CAACCAGATCGGGGCCAAGTT
TUBB3_rv	CCGAGTCGCCCACGTAGTT
MAP2_fw	AGACTGCAGCTCTGCCTTTAG
MAP2_rv	AGGCTGTAAGTAAATCTTCCTCC
NGN2_fw	TGTTCGTCAAATCCGAGACCT
NGN2_rv	CGATCCGAGCAGCACTAACA
SYP_fw	ACCTCGGGACTCAACACCTCGG
SYP_rv	GAACCACAGGTTGCCGACCCAG
SYN1_fw	CCCTGGGTGTTTGCCCAGAT
SYN1_rv	ACCACGGGGTACGTTGTACT
GRIA4_fw	GGCCAGGGAATTGACATGGA
GRIA4_rv	AACCAACCTTTCTAGGTCCTGTG
18s_fw	GTAACCCGTTGAACCCCATT
18s_rv	CCATCCAATCGGTAGTAGCG

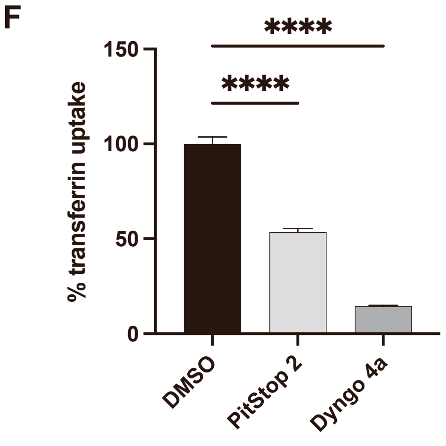

Figure S5 (related to Figure 4): Tau does not mediate its own entry and iPSC-derived neurons express mature markers.

A) Titration of untagged tau assemblies in the presence of 50 nM tau-HiBiT assemblies in HEK-NGL cells and entry quantified after 1 h; n=4. Error denotes SEM.

B) Endosomal lysis assay performed by delivery of NanoLuc-encoding plasmid to HEK293 cells in the presence of excess tau monomer or aggregate (1 μM), adenovirus (MOI 100 IU/cell) or LF (0.2 μl/well); n=3. Error denotes SEM. ** $p < 0.01$ **** $p < 0.0001$ by one-way ANOVA with Tukey's multiple comparisons.

C) Percent positive GFP cells of day 14 GPLN-iNeurons acquired by flow cytometry; n=3, N=3 independent differentiations. 10,000 events minimum recorded. Error denotes SEM.

D) qPCR of neuronal genes at day 0, day 4, day 7 and day 14 of the iPSC differentiation strategy to generate differentiated iNeurons; n=3 from N=3 independent differentiations. Error denotes SEM.

E) Primer sequences for genes targeted in qPCR.

F) Transferrin uptake assay in day 14 iNeurons with 10 μg/ml alexa-fluor-647-conjugated human transferrin. Cells were pre-treated for 1 h with PitStop 2 or Dyngo 4a (both 20 μM) prior to addition of transferrin for 15 minutes. $1x10^4$ events were recorded from 3 independent differentiations. **** $p < 0.0001$ by one-way ANOVA with Tukey's multiple comparisons.

Figure S6 (related to Figure 5)

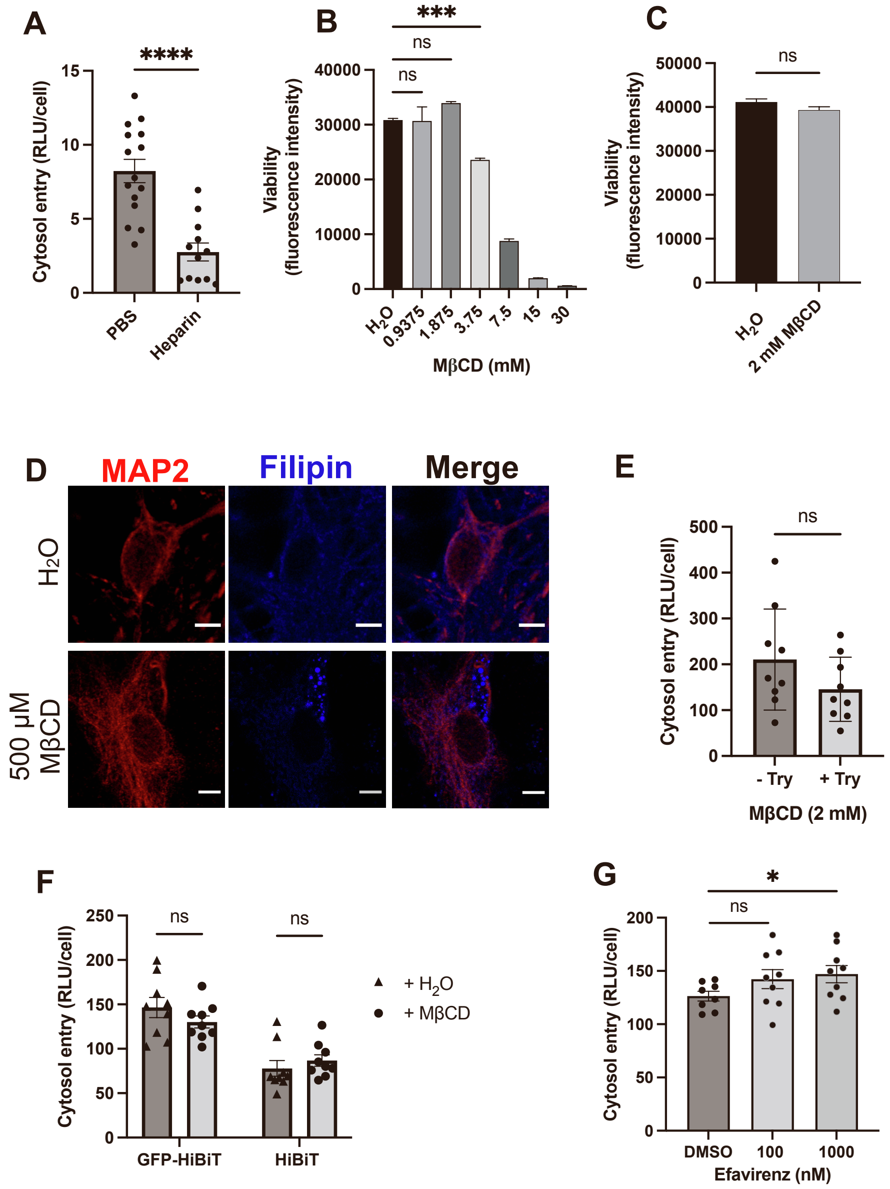

Figure S6 (related to Figure 5): MβCD does not induce cell leakiness and cholesterol-sensitivity is tau specific.
A) Effect of heparin on tau entry in GPLN-iNeurons. Cells were pre-treated with heparin (40 µg/ml) or solvent (PBS) for 1 h prior to assaying the entry of 50 nM tau-HiBiT assemblies in 1 h, n=3-6 from N=3 independent differentiations. Error denotes SEM. **** $p < 0.0001$ by unpaired t test.
B) Presto blue viability assay of GPLN-neurons after 30 minutes pre-treatment with indicated concentration of MβCD followed by a 1 h entry assay; n=3. Error denotes SEM. *** $p < 0.001$ by one-way ANOVA with Tukey's multiple comparisons.
C) Presto blue viability assay of GPLN-neurons after pre-treatment with 2 mM MβCD for 2 h or solvent (water) by a 1 h entry assay; n=6. Error denotes SEM.
D) Total cholesterol staining of DIV 7 primary neurons with Filipin after treatment with solvent (water) or 500 µM MβCD overnight. Scale bars, 5 µm.
E) Effect of trypsin (Try) on entry signal after MβCD treatment. GPLN-neurons were pre-treated for 2 h with MβCD followed by a 1 h entry assay with 50 nM tau-HiBiT assemblies. Cells were then treated with or without Try for 10 minutes at 37ºC and signal acquired; n=3, N=3 independent experiments. Error denotes SEM.
F) Effect of MβCD treatment on the entry of 6xHis-GFP-HiBiT or HiBiT peptide entry alone in GPLN-neurons. Cells were pre-treated for 2 h with 2 mM MβCD or solvent (water) and a 1 h entry assay performed with 50 nM of GFP-HiBiT or HiBiT peptide; n=3 from N=3 independent experiments. Error denotes SEM.
G) Effect of Efavirenz (Efv) on tau entry. GPLN-neurons were pre-treated for 16 h with depicted concentrations of Efv and a 1 h entry assay with 50 nM tau-HiBiT assemblies performed; n=3, N=3 independent experiments. * $p < 0.05$ by unpaired t test.

Figure S7 (related to Figure 6 and 7)

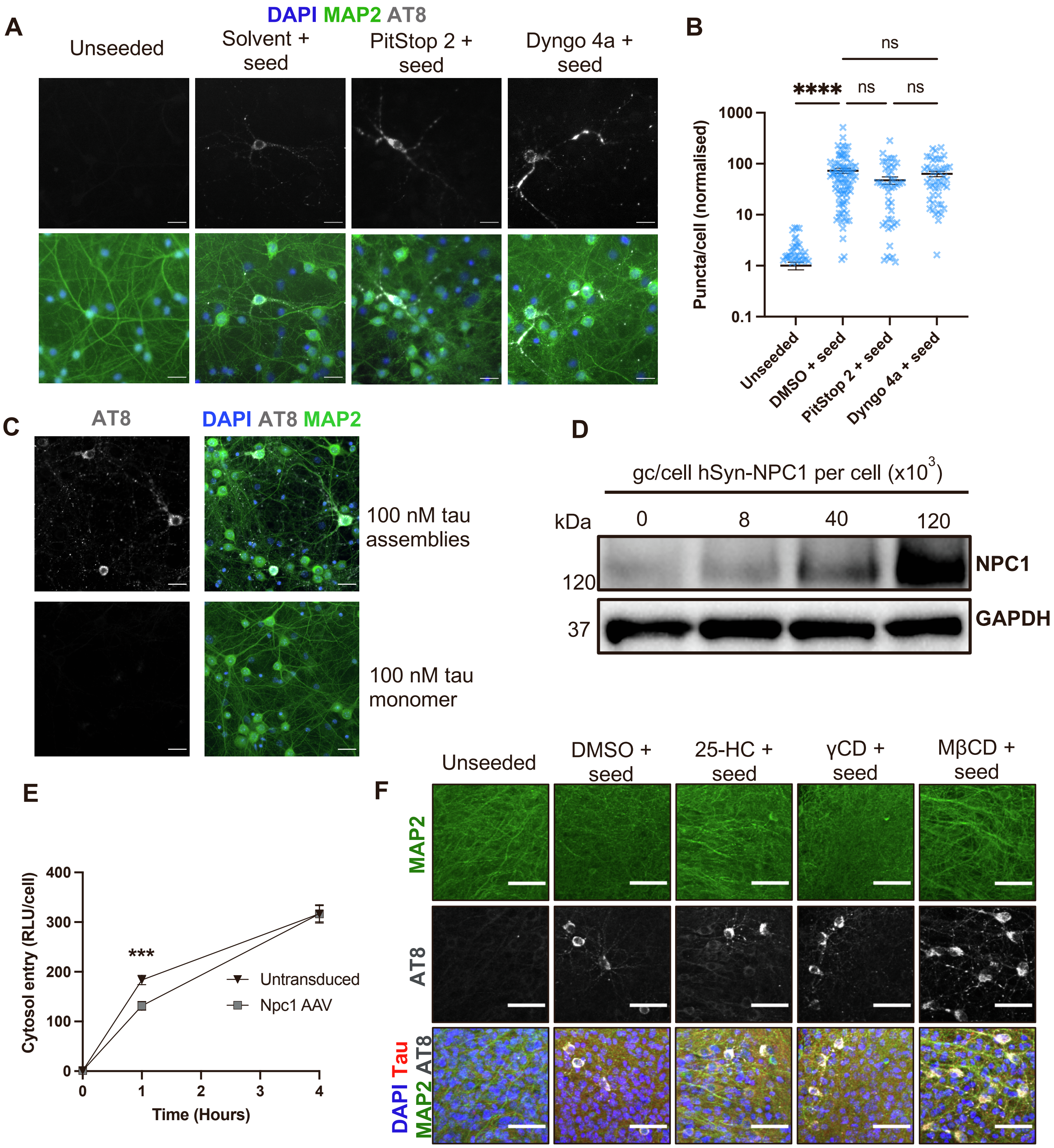

Figure S7 (related to Figure 6 and 7): Endocytosis inhibition does not change seeding and cholesterol manipulation affects neuron entry and slice seeding.

A and B) Fluorescence microscope images and quantification of seeded P301S neurons after pre-treatment with PitStop 2 (20 µM), Dyngo 4a (2 µM) or solvent (DMSO) for 1 h prior to a 7 day seeding assay with 100 nM tau assemblies. AT8 positive puncta were quantified as shown in (**B**); n=3, N=3 independent experiments. Scale bars, 25 µm. Error denotes SEM. **** $p < 0.0001$ by Kruskal-Wallis test with Dunn's multiple comparisons.

C) Fluorescence microscope images of P301S neurons seeded with 100 nM tau assemblies or monomer for 7 days prior to fixation and immunostaining. Scale bars, 30 µm.

D) Western blotting of DIV 7 WT neuron lysates after transduction with AAV1/2 hSyn-NPC1 at depicted multiplicity 96 h prior.

E) Entry of 50 nM tau-HiBiT assemblies to GPLN-neurons transduced with 100,000 genome copies/cell of AAV1/2-hSyn-NPC1 for 96 h prior to assay on DIV 7; n=3, N=3 independent experiments. Error denotes SEM. *** $p < 0.001$ unpaired t test.

F) Fluorescence microscope images of OHSCs quantified in **Fig. 7** (main text). Scale bars, 50 µm.
